# Rapid diffused optical imaging for accurate 3D estimation of subcutaneous tissue features

**DOI:** 10.1016/j.isci.2025.111818

**Published:** 2025-01-23

**Authors:** Shanshan Cai, John Mai, Winn Hong, Scott E. Fraser, Francesco Cutrale

**Affiliations:** 1 Department of Biomedical Engineering, University of Southern California, Los Angeles, CA 90089, USA; 2 Translational Imaging Center, University of Southern California, Los Angeles, CA 90007, USA; 3 Alfred E. Mann Institute for Biomedical Engineering, University of Southern California, Los Angeles, CA 90089, USA; 4 Molecular and Computational Biology Department, University of Southern California, Los Angeles, CA 90089, USA

**Keywords:** optical imaging, cell biology, biophysics

## Abstract

Conventional light imaging in living tissues is limited to depths under 100 μm by the significant tissue scattering. Consequently, few commercial imaging devices can image tissue lesions beneath the surface, or measure their invasion depth, critical in dermatology. We present 3D-multisite diffused optical imaging (3D-mDOI) an approach that combines photon migration techniques from diffuse optical tomography, with automated controls and image analysis techniques for estimating lesion’s depth via its optical coefficients. 3D-mDOI is a non-invasive, low-cost, fast, and contact-free instrument capable of estimating subcutaneous tissue structures volumes through multisite-acquisition of re-emitted light diffusion on the sample surface. It offers rapid estimation of Breslow depth, essential for staging melanoma. To standardize the performance, 3D-mDOI employs customized calibrations using physical tissue phantoms, to explore the system’s 3D reconstruction capabilities. We find that 3D-mDOI can reconstruct lesions up to 5 mm below the surface, requiring ∼300 s of computation time.

## Highlights

• Rapid, non-invasive 3D-mDOI imaging estimates tissue depths up to 5 mm in 300 s
• Consumer-grade OPTIMAP achieves subcutaneous imaging with high computational efficiency
• Hybrid modeling combines optical simulations for accurate sub-surface reconstructions
• 3D-mDOI provides cost-effective melanoma staging via Breslow depth estimation

## Graphical abstract

**Figure:**
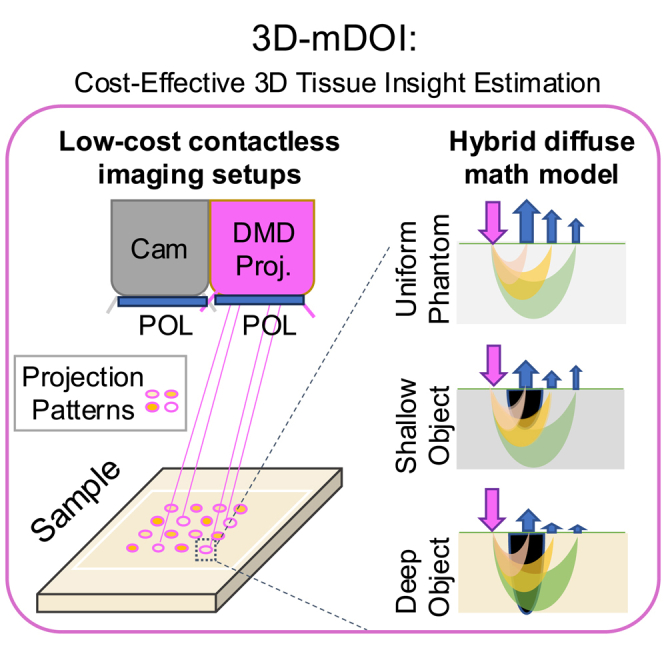

## Teaser

Classification Description: Optical imaging; Cell biology; Biophysics

## Introduction

Current dermatology exams rely on clinician’s ability to deduce abnormal features based on white light reflectance images of the skin surface. Due to the nature of this illumination and the optics involved, this approach is insensitive to features below the surface, necessitating biopsies for histological evaluation of subsurface details by a pathologist.^1^ Recent studies report that a significant number of skin biopsies (23%–44.5%;^2^^,^^3^) are unnecessary or benign. The effectiveness of invasive biopsies is being reconsidered in several medical screening practices, including those for Barrett’s esophagus, ductal carcinoma of the breast, and thyroid cancers.^4^

Current state-of-the-art techniques for dermatologic imaging use reflectance- or fluorescence-confocal microscopy, or optical coherence microscopy.^5^^,^^6^^,^^7^ These approaches can improve diagnoses and reduce unneeded biopsies,^8^^,^^9^^,^^10^ but they have not been widely adopted as a standard for diagnosis, largely owing to the high capital equipment cost, the need for specialized training, and the extended image acquisition times. Therefore, the adoption of these high-performance imaging systems for everyday clinical use remains problematic.^9^^,^^11^ Improving the accuracy of non-surgical approaches, such as optical biopsy, requires developing methods for non-invasively obtaining depth information for moles or other lesions as they invade tissues.

Diffuse optical imaging (DOI)^12^^,^^13^^,^^14^^,^^15^ is a non-invasive imaging technique using the diffusion of near infrared light and photon migration to reconstruct the optical coefficients of a tissue, expressed as the tissue properties of the absorption coefficient $\mu_{a}$, the scattering coefficient $\mu_{s}$. DOI has shown promise in detecting pathologies in the breasts^16^^,^^17^^,^^18^ and the brain,^19^^,^^20^^,^^21^ identifying cancer-specific optical parameters or optical signatures.^17^^,^^18^^,^^22^ Steady-state DOI measures the transmitted and backscattered light resulting from a static light source pointing at a surface,^23^^,^^24^^,^^25^ but faces challenges like instrument’s limits-of-detection, longer acquisition times, and a highly ill-posed inverse model.^26^^,^^27^ Frequency-resolved or time-resolved^28^^,^^29^^,^^30^^,^^31^ measurements can overcome some of these challenges, providing information-rich models, but require increased system and computational costs. Spatial imaging in the frequency domain^32^^,^^33^ employs projected spatial illumination with inverse modeling to provide 2D reconstructions and depth-sensitive maps of the surface optical coefficients of the samples.^34^^,^^35^

DOI is largely limited to 2D acquisition and planar reconstruction, as it relies on light transmittance data.^12^^,^^36^^,^^37^ 3D-DOI has recently been proposed,^38^^,^^39^ but requires *a priori* knowledge of the target objects,^40^^,^^41^ and its reconstructed volumes are limited in size due to computational inefficiencies.^42^^,^^43^ These DOI applications suggest a potential path toward non-contact optical biopsy techniques by leveraging affordable commercial components. Yet, clinical adoption of DOI-based approaches remains limited due to these technical challenges. DOI typically harnesses two mathematical frameworks to describe optical transport in tissues: the radiative transfer equation (RTE), and photon migration via Monte Carlo simulation. RTE simplifies modeling complexity of tissue geometry and homogeneity^23^^,^^44^^,^^45^^,^^46^ to speed up computation, compromising on precision. Decades of tissue photon migration (TPM),^47^^,^^48^^,^^49^^,^^50^ focusing on understanding the diffusion of photons within biological tissues, has led to advanced Monte Carlo modeling of light transport in tissues.^51^^,^^52^^,^^53^ This modeling reconstructs the absorption and scattering properties of tissues below the surface, providing voxel-based insights into tissue health,^54^^,^^55^ but requires substantial computational resources. Hybrid approaches^56^^,^^57^^,^^58^^,^^59^ combine the accuracy of Monte Carlo simulations with the increased computing speed deriving of RTE.

We propose 3D-multisite diffused optical imaging (3D-mDOI) approach representing a significant advancement in non-invasive, contactless, 3D optical biopsy technology. It estimates volumetric maps of tissue properties by leveraging multisite measurements to enhance the quality of 3D reconstructions. Our approach relies on a low-cost imaging platform that integrates a consumer-grade digital projector and CMOS camera to collect diffusion reflectance data simultaneously from multiple surface points building on digital skin rendering techniques.^60^^,^^61^^,^^62^ This innovative system, which remains under a $3,000 budget, is complemented by a hybrid mathematical model that efficiently resolves the complex inverse problem of computing volumetric properties from 2D images. The combination of this platform and model results in a more than 10-fold increase in computational efficiency compared to traditional finite element methods, facilitating the detection and depth estimation of tissue abnormalities. 3D-mDOI’s potential extends to various clinical applications,^10^^,^^48^^,^^63^ offering a new frontier in the diagnosis and study of subsurface tissue volumes.

## Results

### 3D-mDOI

3D-mDOI, enables the reconstruction of unknown geometric features embedded within tissue volumes, based on their optical properties of absorption and scattering, while ensuring a manageable computational runtime. This is achieved through an optimized three steps pipeline: first, image capture using a multisite image acquisition platform; second, 2D nonlinear fitting combined with 3D reconstruction through a hybrid mathematical model; and third, uniformity calibration leveraging experimental reference phantom data. By employing this sophisticated methodology, we enable the effective reconstruction and visualization of the intricate optical property distribution within biological tissues. The synergistic combination of imaging platform and mathematical model enhances the efficacy of 3D reconstruction. The approach requires the selection of a multitude of data points that are treated as 2D diffuse reflectance and analyzed according to the principle of TPM. The analysis estimates the optical properties within individual 3D voxels, ensuring that each voxel is inclusive of detailed and comprehensive information. 3D-mDOI is well posed to facilitate contact-free investigation of sub-surface tissue volumes, offering detection and quantitative depth estimation of areas exhibiting different optical parameters.

**Figure 1:**
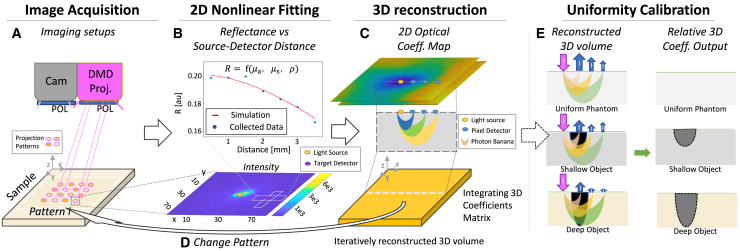
Subcutaneous imaging with 3D-mDOI: approach overview (A–C) The imaging setup consists of a digital micromirror device (DMD) projector that generates patterns of light-beams and a CMOS camera capturing the reflectance of the re-emitted light from the sample. Specular reflection is mitigated by a pair of polarizers (Blue cover), thus improving the camera’s dynamic range for diffuse light. Each captured image is split into (B, bottom) small patches, each centered on a light source, with every pixel functioning as a detector. Reflectance values are selected from a neighboring cross-section (target detector) and associated with a distance from the light source. These values serve as the input for a (B, top) 2D nonlinear fitting of the RTE model, which computes the cross-section’s optical coefficients $\left(\mu_{a},\mu_{s}\right)$ (Magenta Dot). The (C, top) 2D optical coefficient map for each patch is assembled by integrating the optical coefficients from all target detectors. The corresponding (C, middle) 3D photon distribution expands the 2D optical coefficient map into a 3D optical coefficient matrix. We integrate multiple 3D optical coefficient matrices to form a (C, bottom) reconstructed 3D volume by a linear, single-step reconstruction. Each voxel is sampled multiple times, improving the quality of the 3D reconstruction. (D) The projector pattern is systematically scanned over the sample surface, repeating the steps A, B, and C to iteratively update the reconstructed 3D volume. (E) Measurements on a uniform phantom provide a calibration for the reconstructed 3D volume and improving the results. The result is a depth estimation of the ground truth features, evidenced by the visibility of objects or lesions at various depths within the relative 3D coefficient volume.

The multisite acquisition setup, named optical properties tissue imaging multisite acquisition platform (OPTIMAP) (Figures 1A and S3 and Video S1), is specifically designed for high efficiency acquisition of the re-emitted light process. Unlike traditional diffuse optical spectroscopy imaging (DOSI), which relies on lasers and optical fibers, the platform includes a digital micromirror device (DMD) as a light projector, and a near infrared-enhanced camera as a detector array. The DMD creates multiple illumination points, presented as a structured dot-like pattern on the sample surface. To minimize potential confounding light-scattering interference, each illumination point is methodically spaced across sample surface subsections. Incident light coming from the illumination point interacts with the tissue, leading to multiple absorption and scattering events of photons. The camera captures high dynamic range images of the re-emitted light at the tissue-air surface, which forms a diffused halo around each illumination point. Each illumination pattern consists of multiple pairs of an illumination point and its surrounding camera pixels, thereby facilitating a parallel data acquisition process.

Video S1. Demonstration of OPTIMAP in Physical Phantom Imaging This video highlights the synchronized imaging capabilities of the OPTIMAP system. The left panel features the user interface, while the right panel shows the imaging of a PDMS-based tissue phantom designed to replicate dermis optical properties. Initially, the phantom remains in darkness to calibrate the background noise. Upon starting the acquisition sequence, a Digital Light Processor (DLP) projects structured illumination patterns onto the phantom, captured by a high-speed 2 Megapixel CMOS camera with 12-bit pixel depth. The system synchronizes preloaded illumination sequences with data acquisition, enabling efficient and precise capture of light reemission for further analysis

Post-acquisition, the imaging data are processed by a hybrid mathematical model, which effectively integrates elements of the steady-state RTE model^23^ (Figure S1A), with the stochastic realism of Monte Carlo simulation to model the diffusion of light in the tissue (Figures S1B and S1C). Preprocessing of the captured images involves transforming intensity values into diffuse reflectance metrics and calibrating for any instrumental artifacts (Methods). Subsequently, each illumination point is considered a light source, and each pixel of the camera is treated as an individual detector in the hybrid mathematical model. The data from the central detector is grouped with that of its neighboring detectors, with the assumption of quasi-homogeneity for each small tissue surface area. This pooled data are fed to the steady-state RTE model (Figure 1B) to derive the optical coefficients $\mu_{a}\;\text{and}\;\mu_{s}$ for each source-detector pair. This approach enables the rapid calculation of localized optical coefficients with a level of precision tailored to each source-detector pair. The aggregated local optical coefficients for each source-detector pair form a 2D optical coefficient map.

The accelerated reconstruction of the 3D optical coefficients matrix is achieved by transferring the 2D optical coefficient map into a 3D domain, aligning it with a pre-computed 3D photon distribution map (Figure 1C). Photons traveling between a source-detector pair, spaced by a distance ρ, create stochastically characteristic photon trajectories. This characteristic can be described by a 3D spatial probability density function, characterized by a 2D cross-section shaped like a half-moon, and in 3D like a banana.^64^ The central depth of the 3D distribution is approximately half of the source-detector distance. We calculate each partial 3D reconstruction by assigning the $\left(\mu_{a},\mu_{s}\right)$ values for each source-detector pair to its corresponding 3D photon distribution. The intersection portions of the 3D reconstructions are merged by a weighted averaging. Each illumination pattern comprises multiple independent illumination sources, and the final reconstructed volume for an illumination pattern is assembled from these partial 3D reconstructions.

Sequential scanning of multisite illumination patterns across the sample’s entire surface initiates a repeated algorithmic sequence, generating, with each pattern, a new corresponding volume (Figure 1D). The reconstructed 3D volume is computed by averaging the data from the multiple pattern volumes (Figure 1E). This process reduces errors caused by non-uniformities in optical coefficients, which arise from uncertainties in light propagation across varying distances ρ. The resulting volume is then normalized using a calibration matrix from measurements on a featureless phantom. Such uniformity calibration yields a relative 3D coefficients volume, crucial for reducing artifacts due to unevenness illumination of the consumer-grade components in our setup, and consequently enhancing the quality of the 3D-reconstructions. The final output of 3D-mDOI offers an insightful estimation of the embedded features’ depth in the sample. The clarity and precision of this output has the potential to further researchers or clinicians’ understanding of the spatial distribution and relative characteristics of these features or anomalies.

### Validation with simulated tissue phantoms

We demonstrate the 3D-mDOI reconstruction quality utilizing digital tissue phantoms resembling dermatological photo-physical properties, obtaining a range of experimental settings and an estimate of the quality of the 3D-reconstructions. For this purpose, we first synthesize digital phantoms with a variety of features that mimic the reported optical parameters for tissue structures.^48^ The input for the 3D-mDOI process—2D reflectance data at the phantom surface—is estimated using an established Monte Carlo simulation tool,^65^ simulating, within reason, the data being acquired in the multisite image acquisition platform OPTIMAP. We assess the impact of acquisition parameters like sampling frequency and step size on the depth, diversity, and quality of the reconstructions (Note S2, Figure S2). The chosen parameters for the simulations are also employed in the OPTIMAP to ascertain the optimal performance. Subsequently, we visually and quantitatively compare the 3D matrix of optical coefficients $\mu_{a}$ and $\mu_{s}$ computed on the simulated reflectance data of this proposed approach with those from the state-of-the-art finite element method (FEM) simulations.^66^

**Figure 2:**
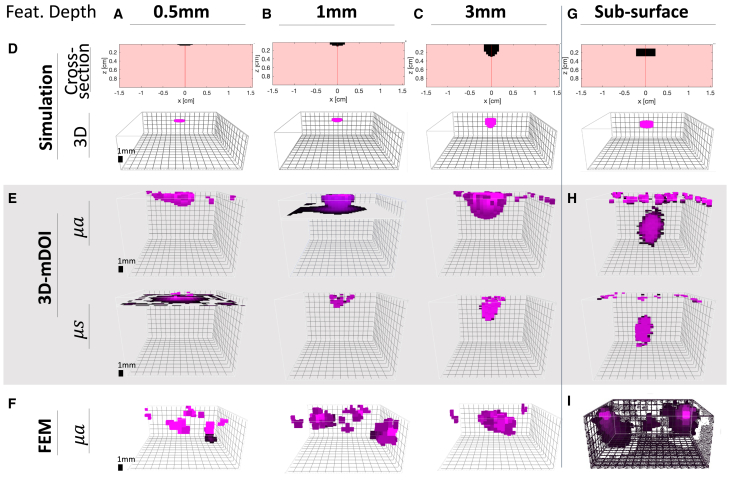
3D-mDOI captures information at various depths in simulated phantoms Comparison of reconstructions using 3D-mDOI and FEM results from numerically simulated phantoms with (A) 0.5 mm, (B) 1 mm, and (C) 3 mm deep features, respectively. (D) XZ cross-section simulations and a 3D rendering of the feature ground truth, as visual references. (E) 3D-mDOI correctly estimates the relative locations of the simulated features in the center of the x-y plane of the volume, correspondingly to ground truth. (F) The FEM reconstructions present incorrect regions for the features. (G) A subsurface feature, invisible from the sample surface, is designed by placing the dye pigment between 1 mm and 3 mm deep. (H and I) The 3D-mDOI reconstruction is at the correct centered position below the surface level in the x-y plane, while (I) the results from FEM analysis are located in multiple incorrect locations.

To visually evaluate our method’s ability, 3D-mDOI is applied to retrieve the location and shape of features at different depths within simulated phantoms (Figures 2A–2C). The testing features are designed to mimic the varying stages of melanoma progression according to Breslow depth,^63^ which is the thickness of a melanoma from the skin’s surface to its deepest penetration point. Specifically, in these digital phantoms we simulate three features that extended from the surface down to depths of 0.5 mm, 1 mm, and 3 mm, respectively. 3D-mDOI’s outputs are compared against both FEM results and the ground-truth simulated phantoms, utilizing the XZ cross-sections as references (Figure 2D). We normalize 3D matrix of optical coefficients, thereby enabling a fair comparison owing to differing dynamic ranges of the results from two methods. The 3D renderings of 3D-mDOI successfully locates the position and size of the single features in the central region of the simulated volume (Figure 2E). As the depth of the features increases, 3D-mDOI accurately reconstructs them as sub-surface volumes, showcasing an improved accuracy in the absorption coefficient matrix as compared to the scattering coefficient matrix. In contrast, results from conventional FEM show the limitations of the approach in these experimental settings, with multiple smaller artifact features outside the expected ground truth region (Figure 2F).

Additionally, we construct a simulated phantom with only sub-surface features (Figure 2D) to assess the performance of 3D-mDOI in scenarios where the sample surface lacks visible information. Despite an increase in surface noise, the results show that 3D-mDOI can reconstruct the hidden feature at the correct location within the simulated data (Figure 2H). In this scenario, the 3D-mDOI reconstructed structures are larger than the features of the simulated phantom. This blurring is the result of an apparent light diffraction effect, attributable to the characteristic shape of the 3D photon distributions, which acts as the point spread functions in the system. Comparatively, the FEM reconstruction recovers multiple features at incorrect positions (Figure 2I).

We evaluate the quality of 3D-mDOI reconstruction using multiple quantitative, rigorous measurements to the same digital phantoms examined in the previous section (Table 1). We compare the performance of reconstructions by 3D-mDOI against FEM across three criteria: intensity-based distance to ground truth, reconstructed image quality, and segmentation error. For the intensity-based distance, we compute root-mean-square-error (RMSE) to assess pixel-wise differences, as well as the Bhattacharyya distance to characterize the similarity in histograms, utilizing the simulated ground truth as reference (Methods). Image contrast, a crucial metric to identify reconstructed image quality, is examined to evaluate the ability of 3D-mDOI to distinguish between different structures or elements in the reconstruction. After these assessments, we perform manual segmentations to analyze the quality of segmentation post-reconstruction. For this latter criterion, we utilize the following metrics to quantify the overlap of the segmented features between reconstructions and ground truth: estimated depth of the reconstructed features, specificity, sensitivity, and dice coefficient. Estimated depth is a metric used to quantify how closely the reconstructed features match the ground truth depth values. Specificity measures the ability of the segmentation to correctly identify true negatives, while sensitivity assesses the ability to identify true positives. The dice coefficient is a measure of the overlap between the segmented features and the ground truth. We then utilize the multi-scale structural similarity (MSSSIM)^67^ to obtain a comprehensive assessment capturing structural information, luminance, and texture facets of the reconstructed volume. When comparing multiple reconstructions, the determination of positively impacting values for these metrics relies on the context of the evaluation. Smaller values are considered advantageous for intensity-based distance metrics such as RMSE and Bhattacharyya distance, whereas higher values are desirable for metrics concerning contrast, specificity, sensitivity, dice coefficient, and MSSSIM. These metrics collectively contribute to a comprehensive assessment of the accuracy, quality, and fidelity of 3D-mDOI and FEM reconstructions when compared to the ground truth data.

**Table 1:** Comprehensive quantitative analysis of synthetic phantom reconstructions

Test Case [mm]	Method	RMSE	Bhattacharyya distance	Contrast	Reconstructed depth [mm]	Specificity	Sensitivity	DICE	MSSSIM
0.5	mDOI	**0.08**	**0.05**	**44.91**	**1.00**	0.99	**0.48**	**0.42**	**0.70**
	FEM	0.21	2.36	0.36	1.50	0.99	0.00	0.00	0.36
1	mDOI	**0.11**	**0.11**	**7.95**	**1.00**	0.99	**0.74**	**0.71**	**0.49**
	FEM	0.26	3.19	0.35	2.5	0.99	0.00	0.00	0.28
3	mDOI	**0.09**	**0.06**	**19.50**	2.75	0.99	**1.00**	**0.74**	**0.69**
	FEM	0.19	1.83	0.93	2.75	0.99	0.32	0.40	0.47
5	mDOI	**0.10**	**0.06**	**16.17**	3.00	0.99	**0.80**	**0.70**	**0.71**
	FEM	0.31	2.99	0.58	3.00	0.99	0.37	0.47	0.31
Sub surface _[1-3]_	mDOI	0.31	**1.19**	**3.51**	3.70–5.80	**0.99**	**0.002**	**0.003**	**0.17**
	FEM	**0.22**	2.89	0.31	**0.00–4.50**	0.96	0.00	0.00	0.00

The reconstruction efficacy of 3D-mDOI and FEM is quantitatively assessed using numerically simulated dermis phantoms with features at depths of 0.5 mm, 1 mm, 3 mm, and 5 mm. This multi-faceted analysis evaluates volumetric reconstructions against a predefined ground truth, encompassing metrics such as pixel-wise distance (root-mean-square-error and Bhattacharyya distance), reconstructed image quality (Contrast) and segmentation accuracy (Feature’s reconstructed depth, specificity, sensitivity, dice coefficient, and multi-scale structural similarity). Superior scores within each sample’s metric are accentuated in bold in the table. The results show that 3D-mDOI outperforms FEM overall. However, both methods exhibit challenges when reconstructing phantoms with subsurface features between 1 mm and 3 mm depth. The advantage of 3D-mDOI, is especially evident in the Bhattacharyya distance, contrast, and segmentation metrics.

Across all evaluated metrics, 3D-mDOI consistently surpasses the gold-standard FEM, outperforming it by 6-fold on average of in the general test cases (Table 1, Test cases 0.5–5 mm). When comparing raw reconstructions, 3D-mDOI presents a lower RMSE and Bhattacharyya values, relative to the ground truth, owing to its improved accuracy in recovering intensity distributions and structural information. Furthermore, results from 3D-mDOI display heightened image contrast, underscoring its enhanced resolution capabilities within its reconstructions. In comparing the segmentation of features, both methods accurately determine the relative orders of dimension of reconstructed depth. 3D-mDOI’s results closely mirror the actual depth, especially for tests where the depth is less than 1 mm. Both methods register high scores in specificity, a result stemming from the feature’s volume being smaller than the background. Segmentations of 3D-mDOI have higher sensitivity scores, indicating a higher proportion of actual feature voxels being identified in the test. This trend is confirmed by higher dice coefficient of 3D-mDOI, showing better alignment of segmented region to the expected ground truth. Lastly, the elevated MSSSIM score for 3D-mDOI testifies to its improved reconstruction quality across human observers’ perception.

We undertake additional quantitative comparisons with a subsurface feature ranging between 1 mm and 3 mm in depth (Table 1, Subsurface test case). The visual interpretation of the reconstruction of this fully embedded feature indicates the superior accuracy of 3D-mDOI over FEM (Figure 2), supported by further quantitative measurements. In this scenario of a subsurface feature, 3D-mDOI presents a general 68% decrease in the performance metrics, when compared to the other test cases with features extending from the surface into the tissue (Table 1, Test cases 0.5–5 mm). This general degradation of results is due to a combination of reduced amount of signal and a blurring effect due to the shape of the point spread function of the system.

### Optical coefficient validation with physical phantoms

**Figure 3:**
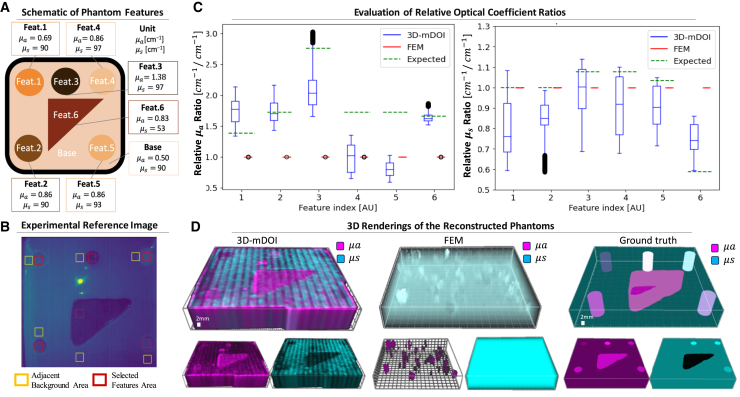
3D-mDOI reconstructs distinguishable sub-surface features in the physical phantom Comprehensive analysis evaluating the physical phantom reconstruction for the methods of 3D-mDOI and FEM. (A) A physical phantom, crafted with different proportions of titanium dioxide (TiO2) and India ink in a polydimethylsiloxane (PDMS) medium, contains six unique features with varying absorption ($\mu_{a}$) and scattering ($\mu_{s}$) coefficients. The multisite image acquisition platform facilitates systematic scans of the phantom, employing a digital micromirror device (DMD) to create specific illumination patterns and a CMOS camera to capture the re-emitted light. (B) In the experimental reference intensity image, regions in red bounding boxes are selected features, which are subsequently normalized by the regions of background in neighboring yellow bounding boxes. Boxplots show the relative optical coefficients ($\mu_{a}$, $\mu_{s}$) ratio for phantom features, with the distributions of approximately $10^{5}$ voxel samples for each feature. (C) These plots provide a clear statistical representation where the central box spans from the first quartile to the third quartile, bisected by a line representing the median. The whiskers extend to a maximum of 1.5 times the inter-quartile range, while any data points beyond these whiskers are denoted as flier points. $\mu_{s}$ results from 3D-mDOI largely coincide with the expected optical coefficient ratios of the features. However, certain deviations can be observed in the ratio of $\mu_{a}$, especially for feature 4 and 5. Comparably, the FEM results have limited dynamic range, obscuring distinct differences between feature values. (D) 3D renderings of the phantom showcasing the reconstructed features and highlighting 3D-mDOI’s robustness against experimental noise compared to the FEM’s more ambiguous renderings with respect to the ground truth.

We utilize the OPTIMAP to perform 3D-mDOI on physical tissue phantoms containing 3D-molded features with different absorption ($\mu_{a}$) and scattering ($\mu_{s}$) coefficients. These phantoms are crafted with titanium dioxide (TiO2) and India ink embedded in a polydimethylsiloxane (PDMS) bulk medium with different ratios, in order to change the absorption and scattering properties. The expected optical parameters for six different features in the physical tissue phantom (Figure 3A) are computed based on the ratios of chemicals added in each feature (Table S1, Part 1).

To assess the performance of both 3D-mDOI and FEM, we quantify the relative optical coefficients ($\mu_{a}$, $\mu_{s}$) of the phantom features and visually validate these findings through 3D renderings. First, we perform corrections for instrument uneven illumination by calculating the relative optical coefficients ratio for each distinctive feature. To accomplish this, we normalize the feature data (Figure 3B, Red boxes), utilizing the values from the adjacent backgrounds (Figure 3B, Yellow boxes). We illustrate the comparison of the relative optical coefficients ratio of the features derived from both the 3D-mDOI and FEM methods (Figure 3C) to delineate the distribution of results, pinpointing details such as the median, potential outliers, and any discernible skewness in the data. The results from 3D-mDOI for relative scattering coefficient ( $\mu_{s}$ ) ratios generally align with expected feature values, while those of the relative absorption ( $\mu_{a}$ ) ratios are reasonably estimated ∼66% of the times, owing to two features 4 and 5, that display deviations from the expected value. The 3D-mDOI method presents greater variance in the reconstructed relative optical coefficients when compared to FEM owing to the FEM results being an almost constant value of 1, negating distinctions between features in the plot.

To provide a visual insight into these results, we normalize the reconstructed optical coefficients of the two approaches to a comparable dynamic range. 3D renderings of the 3D-mDOI reconstruction (Figure 3D) clearly enables visualization of the tissue phantom features, suggesting the method’s resilience against consumer-level instrumentation and its associated experimental noise. FEM, on the other hand, offers an imprecise representation of the features in its normalized $\mu_{a}$ results, while its normalized $\mu_{s}$ output remains static at $\mu_{s}=1$ , lacking sufficient numerical depth and insight.

### Feature depth estimation with physical phantom

We implement an additional step in 3D-mDOI to understand and parametrize its depth estimation of features in physical phantoms. To study the inherent constraints of the OPTIMAP’s capacity for depth estimation, we conduct an in-depth analysis using an experimental uniform phantom (Note S3, Figure S5). This analysis focuses on the relationship between the signal-to-noise ratio (SNR) of the re-emitted signal and the 2D RTE fitting error, observing how this error escalates as the detector’s position increasingly deviates from the center of illumination (Figure S5). As the source-detector distance extends to 10 mm, the SNR of the signal drops from 41dB to 11dB, and the reflectance fitting quality drops sharply. This result signifies that the system’s maximum reconstruction depth, with the setup utilized in this work, is constrained to approximately half that distance, or 5 mm.

**Figure 4:**
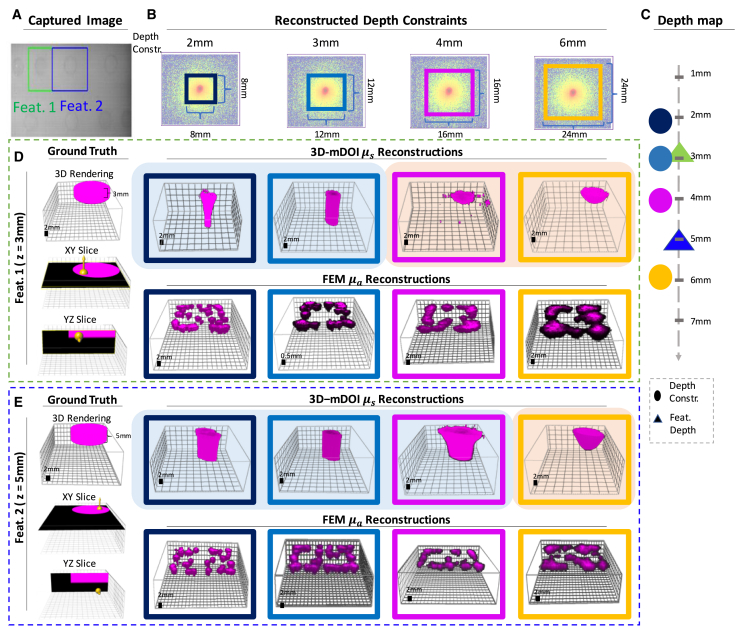
Depth estimation using 3D-mDOI and the significance of optimal region of analysis selection (A–C) The sampling area size significantly influences the accuracy of the 3D-mDOI reconstructions. We acquire images with a large field of view of a physical phantom containing surface-level features with optical properties different from the bulk. We select (A) two distinctive features (feature 1 = 3 mm, feature 2 = 5 mm deep) for this analysis. The fidelity of reconstructing the features at various depths is analyzed utilizing (B) multiple regions of analysis (ROA). These ROAs restrict the surface pixels utilized for analysis, defining the reconstruction depth. Based on the diffusion model in this work (Note S1), the depth of 3D-mDOI reconstruction is limited to a quarter of the length of the ROA side. The depth map (C) shows the relationship of the depth constraints with the features’ depth. (D) Analysis results for feature 1 (depth: 3 mm). A shallow depth constraint, shorter than the actual depth of the feature (D, Navy, and Blue), leads to reconstructed objects that extend to the phantom’s base. Conversely, when the depth constraint exceeds the feature’s actual depth (in this case 3 mm) (D, Magenta, and Yellow), the depth estimation of the reconstructed object improves. (E) Reconstructions of feature 2 (depth: 5 mm), present a similar trend, with a marked shift in the depth profile as the ROA-induced depth constraint changes from 4 mm to 6 mm. The figure underscores 3D-mDOI’s inherent ability to discern object depths by strategically adjusting the analysis ROA, a capability absents in conventional finite element method.

Building on OPTIMAP’s depth constraint, we evaluate various region of analysis (ROA) (methods) candidates to counterbalance the increased experimental noise observed in the data from physical phantoms compared to synthetic ones (Figure 4). The example in (Figure 4A) shows two features which are 3D-molded (Figure S6) with a round subsurface shape (Table.S1, Part 2) in a physical tissue phantom and that are respectively 3 and 5 mm deep. The selection of the ROA is theoretically connected to the quality of depth estimation (Methods). The proper choice of ROA size includes the necessary data for reconstruction while filtering out data having low SNR, leading to better depth estimation outcomes of 3D-mDOI.

For a comprehensive exploration, we first study two extreme cases where the depth constraints are set at 1 mm and 8 mm, under and oversampling depth, defining the minimum and maximum ROA thresholds in our experiment (Figure S7). Specifically, excessively expansive ROAs (Figure S7C Brown) are prone to undesirable crosstalk from neighboring illumination sources, accompanied by noise infiltration from aberrant pixels. Conversely, an overly constrained ROA (Figure S7C Purple) captures insufficient portion of the photon distribution’s shape, which is imperative for a proper 3D reconstruction. ROA regions that deviate significantly from an optimal size produce 3D-mDOI reconstruction with evident challenges. In the subsequent phases of our analysis, optimal ROA are estimated from experimental settings and utilized for further analysis.

Following these boundary cases, we utilize ROA with intermediate dimensions in 3D-mDOI to evaluate the inherent depth estimation of features, revealing the relationship between reconstruction depth and sampled area, a characteristic not mirrored in the standard FEM (Figure 4). We execute four separate experiments, establishing depth constraints of 2, 3, 4, and 6 mm respectively (Figure 4C). For ROAs areas between 4 × 4 mm to 12 × 12 mm, the depth constraint ranges from 1 to 3 mm, shorter than the true depth of the feature 1 (3 mm). In these cases, the objects in the reconstructed volume are ill-informed and the reconstructed objects present an artifact, appearing to extend to the bottom boundary of the phantom (Figure 4D, Navy, Blue). Expanding the fitting ROA area increases the pixel count which consequently enhances the SNR. Larger ROAs over 12 × 12 mm yield improved 3D-mDOI images with a sampling depth exceeding 3 mm, outperforming the depth of feature 1 and leading to more accurate depth estimations (Figure 4D Magenta, Yellow). The similar trend is observed for feature 2 (Figure 4E). Errors in the reconstructions are noticeable in ROA areas smaller than 24 mm × 24 mm (Figure 4E, Navy, Blue, Magenta), while reconstructions begin to show a finite depth for feature 2 indicating a depth range of 4 mm–6 mm (Figure 4E, Yellow). The study reveals the potential for accurately estimating object depth by optimizing the ROA area, a characteristic not exhibited by conventional FEM.

## Discussion

3D-mDOI delivers subsurface tissue imaging by synergistically integrating a non-invasive, consumer-friendly imaging system with an advanced analytical method. The OPTIMAP (Figure S3) integrates a DMD for pinpoint illumination and a CMOS camera for non-contact detection, increasing the richness of data. Analytically, 3D-mDOI merges the RTE with Monte Carlo simulations (Note S1), balancing model accuracy with computational efficiency (Note S4). Its structured illumination approach, coupled with advanced analysis, effectively leverages photon distribution oversampling to ensure comprehensive information on each voxel, reducing the likelihood of reconstructive errors. Rigorous calibration protocols address potential pitfalls such as inconsistent background light, intrinsic acquisition noise and uneven illumination. Together, these features enable 3D-mDOI to deliver consistent and reliable 3D analyses of tissue composition.

Our approach outperforms the conventional modeling techniques in the reconstruction of simulated tissue phantoms, an advancement quantified by both visual (Figure 2) and numerical assessments (Table.1). The 3D-mDOI achieves up to 40 times greater image contrast, reduces RMSE by 60%, decreases the Bhattacharyya distance of histogram distribution by 97%, and enhances the reconstruction accuracy of subcutaneous structures by 85%. This heightened performance is attributable to our approach of treating each pixel as an independent detector, combined with the utilization of a large set of spatially constrained measurements which are guided by a 3D probability distribution function. As a result, the reconstructed voxels are informed from multiple angles and multiple measurements, significantly elevating the level of precision.

It is important to highlight that the performances of both the conventional and 3D-mDOI methods tend to diminish for test cases featuring lower photon information, particularly in simulated tissue phantoms with subsurface features. Certain performance metrics, specifically RMSE and reconstructed depth, indicate a potential underperformance of 3D-mDOI compared to FEM when assessing some subsurface features (Table.1). The elevated RMSE for 3D-mDOI, for this type of feature, stems from a mismatch between the depth estimated by 3D-mDOI (3.7–5.8 mm) and the actual depth range (1–3 mm). While the FEM analysis boasts an RMSE that’s 33% lower than that of 3D-mDOI—attributed to its depth estimation (0–4.5mm) aligning more closely with the true range—it falters in accurate XY positioning, resulting in null values for both the Dice coefficient and MSSSIM. Despite the challenges posed by this subsurface feature, especially the reduced photon reflection, 3D-mDOI consistently outperforms FEM across most evaluation metrics in simulated tissue phantoms.

The OPTIMAP system, with its innovative non-contact design, brings a transformative approach to the acquisition of diffuse optical imaging data of physical phantoms, despite a decrease of the signal-to-noise ratio (SNR). This reduction in SNR presents a manageable challenge within the robust framework of RTE nonlinear fitting, such as increasing the reconstruction crosstalk between the two optical coefficients. However, this aspect has been effectively addressed in the majority of our 3D-mDOI reconstructions (Figure 3), exhibiting robust performance, accurately reflecting the expected $\mu_{s}$ values and capturing 66% of the expected $\mu_{a}$ values. Rendered in 3D, the reconstruction successfully highlights tissue phantom features, showcasing the method’s potential despite the variability introduced by experimental noise and consumer-level instrumentation. In contrast, the FEM yields consistently uniform values, offering limited detail and failing to distinguish between different features effectively. 3D-mDOI further improves by 8-fold computational memory efficiency with a 43-fold reduction in computational time when processing physical phantoms (Note S4, Figure S8).

Our processing pipeline incorporates additional constraints to mitigate the challenges of reconstruction’s fidelity as depth increases in physical phantoms (Figure S4). By imaging a uniform phantom, we experimentally determine the absolute maximum reconstruction depth of the OPTIMAP system (Figure S5). Analytically, we constrain the region of analysis to understand the effects on reconstruction quality (Figure 4). Our results suggest that features extending deeper than the OPTIMAP sampling will appear as extending to the phantom’s bottom edge. This is an expected result, as 3D-mDOI in this case would not have the information necessary for determining the deepest point of such feature. However, this observation also unveils the promising prospect of honing the depth estimation of biological features through a specific selection of the region of analysis, an attribute not paralleled in traditional FEM techniques. Refining our methodology to achieve precise depth estimation of features represents a key direction for our future research.

3D-mDOI approach offers a promising avenue for improving the quality of information of diffuse photons information by harmonizing advanced hardware with innovative computational algorithms. The OPTIMAP is designed with two essential features in mind: cost-effectiveness, achieved by using consumer-grade electronics, and patient comfort, ensured by limiting data collection to manageable durations. These choices, however, entail a lower signal-to-noise ratio for the reflectance data in the deeper sections of the testing samples, limiting the accuracy of the reconstruction depth-wise to approximately 5 mm in this current setup. Improvements in detection depths for 3D-mDOI are intrinsically tied to higher illumination power of the projector or to a higher sensitivity of the camera instrument. While these technical specifications generally correlate with the device costs, it should be noted that recent advancements in manufacturing have introduced high-sensitivity tools in commonly used consumer technologies.^68^ For retaining a budget-friendly hardware architecture, deep learning algorithms have proven valuable in bridging the gap between lower cost detectors and high quality data, for example through denoising.^69^ Integration of these deep learning techniques is particularly advantageous in scenarios involving hardware limitations or external noise sources, extending the 3D-mDOI’s robustness and applicability in diverse conditions.

3D-mDOI has promising applications in optical biopsy and melanoma detection, offering sub-millimeter spatial resolution crucial for assessing Breslow depth in skin cancer. With the healthcare industry’s increasing demand for cost-effective imaging, this technique provides faster acquisition and improved patient care. As the system continues to refine its reconstruction accuracy in future iterations, its scope is poised to expand, potentially catering to the diagnostic requirements of multiple pathologies in a broader array of tissues, beyond the skin, such as in dental, breast, and brain.

### Limitations of the study

The OPTIMAP system, with its innovative low-cost and non-contact design, introduces an innovative approach to optical mapping. This design choice, while facilitating accessibility and ease of use, presents a nuanced challenge in achieving high-quality estimations at greater depths. The system’s sensitivity to signal-to-noise ratios is a critical aspect of its operation. Specifically, the re-emitted light’s low signal-to-noise intensities, compounded by instrumental noise, naturally confine the system’s effective reconstruction depth to approximately 5 mm. This limitation is not a flaw but rather an area for future enhancement.

The reconstruction reliability subtly diminishes with increasing depth. This phenomenon is attributed to the intrinsic behavior of photon migration, wherein photons traveling to deeper regions of a sample encounter greater energy loss. Consequently, the likelihood of capturing re-emitted light from these regions decreases, leading to a shift in optical values as a function of depth. This effect inherently challenges the 3D-mDOI’s capability to accurately estimate finer feature depths.

The inherent properties of photon migration with depth pose challenges for analyzing subsurface features (Note S6, Figure S10). While light intensity decreases exponentially within the sample, leading to smaller perturbations in reflectance for deeper features, the “banana” shape of the 3D photon distributions widens with depth. These effects result in blurred and over-estimated reconstruction of the deeper samples, affecting the performance of 3D-mDOI. A potential improvement is to incorporate 3D-mDOI with other imaging modalities, such as time-resolved measurements^70^ or wavefront shaping,^71^ to achieve a thinner or more constant and simple 3D photon distribution along the z axis. However, these solutions significantly increase the cost of the imaging device, necessitating a careful consideration of the tradeoff between cost and precision based on application requirements.

However, it is important to recognize that the system’s current limitations open avenues for methodological innovations. For instance, identifying and targeting the optimal region for analysis could significantly enhance object segmentation, effectively mitigate some of the outlined challenges. The pursuit of advancements such as depth-based correction methods presents a promising direction for future research. These improvements hold the potential to refine the system’s accuracy in finer depth estimation, further solidifying the OPTIMAP system’s position as a valuable tool in the field of optical mapping. This perspective underscores the system’s current achievements while highlighting the constructive path toward its technological evolution.

The reconstruction process offers opportunities for improvement stemming from solving the RTE inverse problem. The intrinsic non-uniqueness nature of the RTE problem leads to reconstruction crosstalk between absorption coefficients $\mu_{a}$ and scattering coefficients $\mu_{s}$. Notably the $\mu_{a}$ values in the tissue are significantly lower, approximately 100-fold less, than the $\mu_{s}$ values. This physical discrepancy emphasizes the potential for estimation inaccuracies for $\mu_{a}$, underscoring the need for improved solutions. A promising approach to mitigating the crosstalk issue involves the implementation of a predictive normalization strategy within the fitting model. Such strategy could harmonize the interplay between $\mu_{a}$ and $\mu_{s}$ enhancing the accuracy of estimations. An additional challenge relates to the system’s reliance on pixel-level fitting that, while instrumental in extracting subsurface information, concurrently introduces noise and grid artifacts. These artifacts, though manageable, necessitate proper adjustment of visualization thresholds to omit simple, small clusters at the sample’s top layer.

When transitioning 3D-mDOI techniques from simulations and physical phantoms to clinical data, the system faces additional challenges. OPTIMAP needs improvement from the current research prototype to a clinically ready device, ensuring the stability and reliability of data collection (Note S5, Figure S9). An adaptive imaging protocol covering various skin and lesion conditions must be developed to tackle complex clinical and biological variations, including skin type, tumor metabolism, and experimental noise. Since 3D-mDOI’s reconstruction accuracy is highly related to the quality of multi-stage calibration, adjusting preprocessing and calibration steps based on clinical data will require initial validation with patient data and additional computational time before clinical use.

## Resource availability

### Lead contact

Further information and requests for resources should be directed to and will be fulfilled by the lead contact, Francesco Cutrale (cutrale@usc.edu).

### Materials availability

The physical phantoms are available from the corresponding author upon reasonable request.

### Data and code availability

The manuscript reports previously unpublished custom code that is central to supporting the achievements in this work.

• The relevant demo data and instruction have been deposited on Github at https://github.com/shanshan3333333/3D-mDOI. (https://doi.org/10.5281/zenodo.12687294).
• The relevant demo code and instruction have been deposited on Github at https://github.com/shanshan3333333/3D-mDOI. (https://doi.org/10.5281/zenodo.12687294).
• Any additional information required to reanalyze the data reported in this paper is available from the lead contact upon request.

## Acknowledgments

This work is supported by 10.13039/100013829Alfred E. Mann Institute for Biomedical Engineering University of Southern California.

## Author contributions

S.C. acquired, analyzed the results and wrote the code. S.C. and F.C. provided conceptualization. S.C., J.M., and F.C. helped in the experimental design. S.C. and F.C. acquired data. S.E.F. provided supervision. S.C., J.M., and F.C. wrote the paper. W.H. supported review and editing.

## Declaration of interests

The University of Southern California has filed a provisional patent application covering this method listing S.C., S.E.F., and F.C. as inventors.

## STAR★Methods

### Key resources table

**Table:** 

REAGENT or RESOURCE	SOURCE	IDENTIFIER
**Chemicals, peptides, and recombinant proteins**		

Polydimethylsiloxane (PDMS)	Dow, America	Sylgard 182
Titanium Dioxide (TiO2)	Sigma-Aldrich, America	CAS#1317-70-0
India Ink	Higgins, America	

**Software and algorithms**		

Source Code, Demo and Data	https://github.com/shanshan3333333/3D-mDOI	https://doi.org/10.5281/zenodo.12687294

### Method details

#### 3D-photon distributions and look-up map generation

In our work we employed a 3D Monte Carlo simulation^65^ to model photon propagation within a tissue phantom, reflecting specific parameters indicative of melanoma characteristics. The constructed phantom, designed to mimic homogeneous dermis tissue, had dimensions of 30 × 30 × 10 mm with a voxel size of 0.5 × 0.5 × 0.2mm, aligning with melanoma’s typical size and Breslow depth. The simulation utilized a single collimated Gaussian light beam as the incident light source, with a 680nm wavelength, within the red region of the visible light spectrum. Although the light beam’s waist size (0.01mm) is relatively small compared to the voxel size of the phantom, its central position within the phantom was designated as the incident light’s point of entry. The phantom’s air-tissue interface voxel were considered as detectors.

The photon trajectories computed by Monte Carlo simulation were grouped based on their exit point at the air-tissue interface. By aggregating the trajectories with identical entry and exit voxels, the resulting map represents photon distributions in three dimensions. Each voxel’s photon trajectory was normalized by the total number of incident photons. To account for the inherent stochastic nature of the Monte Carlo method, we executed this simulation five times, yielding an averaged 3D photon distribution map. This process facilitated the computation of the reflectance ratio, determined by normalizing the count of photons escaping at specific locations against the total number of incident photons. This ratio is indicative of the degree of signal loss in 3D back-projections and offers important correction weights for the 3D-mDOI’s reconstruction stage.

#### Simulated phantom design

In our effort to evaluate the performance of 3D-mDOI, we engineered multiple simulated tissue phantoms incorporating vatious pigment-like features (Figures 2 and S2). These standardized phantoms, measuring 30 × 30 × 10 mm, were segmented into two distinct types of tissue: the uniform dermis and the embedded pigment-like features. Each tissue type, owing to its specific composition encompassing blood, water, fat, and melanin concentrations, exhibits unique optical properties as derived from the Jacques et al.'s tissue simulation model.^72^ For the skin pigment simulations, we utilized the melanin concentration as a mean to drive the optical properties. Specifically, we set a melanin volume fraction of 0% for dermis tissue and 20% for the pigment feature. The absorption coefficient ($\mu_{a}$) for the dermis and the embedded features is determined to be 0.04 mm^−1^ and 4.879 mm^−1^ under red light (680nm), respectively.^65^ Contrarily, the scattering coefficient ($\mu_{s}$) remains consistent at 29.411 mm^−1^ for both the dermis and embedded features, regardless of melanin fraction variations.^65^ To further vary the features with different embedded depth, we designed two distinct inclusion designs for these embedded features: surface protrusions and subsurface inclusions. The surface design encompassed semi-spherical features, 2mm in radius, progressively embedded at depths of 0.5, 1, and 3mm, informed by Breslow depth.^63^ In contrast, the subsurface design introduced cylindrical inclusions, with depth spanning from 1mm to 3mm and a consistent 2mm radius, epitomizing 3D-mDOI’s capability to reconstruct subsurface objects.

We used simulated phantoms to benchmark both visual and quantitative performance (Table 1) and to emulate the image acquisition phase (Figure 1) through Monte Carlo simulation via photon migration.^65^ This process mirrored structured illumination by altering the location of the illumination point, yielding a spectrum of reflectances across the heterogeneous tissue model. Apart from the the incident light location, the parameters employed for the Monte Carlo simulation remained consistent with those defined for the 3D-photon distribution look-up map, ensuring methodological consistency and data accuracy. The computed reflectance data informed the 2D nonlinear fitting components of 3D-mDOI.

#### Tissue phantom construction

We develop representative physical phantoms utilizing materials known to emulate human tissue’s optical coefficients.^73^ We used Polydimethylsiloxane (PDMS, Sylgard 182 silicone elastomer, Dow, America), owing to its consistent behavior, stability, and tissue-like optical characteristics. Changes in the scattering ($\mu_{s}$) were achieved with the addition of TiO_2_ (TiO_2_, CAS#1317-70-0 Sigma-Aldrich, America), while the absorption ($\mu_{a}$) was changed using India Ink(Higgins, America). Adjusting the proportions of TiO_2_ and India Ink within the PDMS, we engineered phantoms that replicated the optical coefficients of human tissue and moles.^65^ The dynamic range of the optical properties for the physical phantom is designed to both be possible to construct with typically utilized materials (India Ink, TiO2) but also to be sufficient for evaluating the sensitivity limitations of 3D-mDOI in OPTIMAP experiments. We designed two types of phantoms: Phantom A (Figure 3) with surface-level features, and Phantom B (Figure 4) focusing on sub-surface features, each designed to evaluate 3D-mDOI’s prowess in reconstructing optical parameters across depths. Further details on the features' optical parameters are detailed in Table S1.

In our approach to craft multi-feature phantoms, we utilized 3D printed molds, ensuring precision and repeatability. Utilizing polylactic acid as the mold material (Figure S6), we developed a process to effectively mitigate bubble formation. We embedded various solid features within a 3D-printed lid as our mold structure, and casted a PDMS base beneath it. Post hardening of the PDMS base, this lid was detached and the resulting cavities were filled with a precisely tuned mixture of PDMS, TiO2, and India Ink, to achieve specific optical coefficients contrast from the PDMS background. The crafting of Phantom B introduced an extra layer, where a 1mm bulk PDMS overlay ensured complete encapsulation of the features. To address the challenge of bubble formation, each PDMS layer underwent vacuum chamber degassing. While this step significantly reduced the presence of bubbles, we acknowledge the potential inclusion of microbubbles and minute non-uniformities in the chemical mixture, which might introduce variability in the optical parameters' readings (Figure 3D).

#### Experimental image acquisition

The cost-effective and non-invasive reflectance data acquisition was made possible by our innovative Optical Properties Tissue Imaging Multisite Acquisition Platform (OPTIMAP), composed of readily available consumer-grade equipment. The platform integrates a CMOS camera (acA2000-340km, Basler ace, Germany) and a programmable digital light projector (DLP4500-C350REF, Texas Instruments, America), positioned approximately 30 cm from the sample and roughly 15cm apart from each other. The DLP projects an array of dot-like patterns onto the sample surface using 630nm red LED light, while the CMOS camera simultaneously captures the light re-emitted at the tissue-air interface. We employed optical cross-polarization on both the camera and DLP to effectively suppress any specular reflectance. The total expenditure for this setup was approximately $3,000, offering futher prospects for cost-reductions with future enhancements.

We designed an acquisition protocol to ensure the automated collection of high-quality re-emitted light data. A custom C program synchronized changes in the DLP’s patterns with the camera operations, streamlining the data acquisition process. To bolster the signal-to-noise ratio, particularly for pixels further from the light source, we captured 50 images for each illumination pattern, utilizing the same acquisition settings, and averaged the data. Additionally, we created an ultra-high dynamic range (UHDR) output by acquiring images across multiple exposure times for each pattern, such as the samples in Figure 4 that spanned exposure times of 10, 20, 40, 80 and 160 ms

The conventional HDR technique merges several 8-bit images captured at varied exposure settings to yield an image with a superior dynamic range. We refined MATLAB’s 'makehdr' function to enable output in a 16-bit image format. The optimal UHDR setting was determined by balancing the dynamic range of the resultant UHDR images and the acquisition duration of the raw images (Figure S3). Eventually, the UHDR image, derived from raw data with exposure times of 10, 20, 40, and 80, was employed to assess the efficacy of 3D-mDOI when working with physical phantoms.

#### Preprocessing of imaging data

To ensure reliable reconstruction fidelity, multi-stage calibration for background illumination, camera noise, and illumination unevenness are necessary for the data captured from optical multisite image acquisition platform (OPTIMAP). Background noise during acquisition predominantly arises from light leakage in the DMD projector, ambient room light, and inherent camera noise. To address this, we captured a background image with all the DMD mirrors turned off. This image, which represents the static noise components, was then subtracted from the experimental images, eliminating constant background noise from the DMD projector. Additionally, camera noise was minimized by performing a black correction for each exposure setting by capturing 50 dark frames for each exposure time and averaging them to obtain an exposure-dependent correction frame. Subsequently, the experimental data were adjusted by subtracting the corresponding dark frame based on its exposure duration. We maintained a constant gain, collecting datasets after camera reached temperature stability to minimize the acquisition variations during the experiment.^74^ For more uniform illumination, a normalized flat-field correction matrix was generated by imaging white targets at varied exposure durations. During the experimental acquisition phase, we utilized the pre-stored flat-field correction matrix to preprocess the captured images.

Signal-to-Noise Ratio (SNR) is metric for quantifying the amount of photon information captured by our acquisition platform. This metric, expressed in decibels, measures the proportion of the desired signal to the background noise. We utilize the average intensities of the detected signal (${I_{\rho }}_{.}$) at a fixed source-detector distance ρ against the average intensities of the bottom 5% intensities as the background ($I_{B^{\prime }}$). This approach allows for a standardized assessment of OPTIMAP’s capability to discern signal from the background noise.

(Equation 1) $$SNR=20\times \log_{10}\left(\frac{I_{\rho }}{I_{B^{\prime }}}\right)$$

We convert the experimentally measured intensities to reflectance for each illumination point. We assume each illumination point had minimal cross talk with others, covering a finite Region of Interest (ROI) in a captured image, thanks to our systematically designed illumination pattern. This premise allows us to crop the acquired images into smaller patches, each containing just one illumination point. Based on reflectance data in captured multisite image acquisition platform with a singular illumination point projected onto the test, we pre-determined the size of the ROIs. Specifically, we defined the ROI size by identifying the broadest range that encompassed pixels with positive intensity after background noise subtraction. During the experiments, we extracted local maximum pixels from the data, identifying them as illumination point locations. Intensities in each patch were later normalized by the local sum. This intermediary step yields a value close to the ratio of detected reflectance light in a pixel to the radiance from a specific light source in the hybrid mathematical model (Note S1). The correction parameters utilized in this preprocessing step are determined through an optimization process from experimental data of a uniform calibration sample. In practice, we determined the best scaling and offset values by using a least squares fitting method to linearly represent relationship between the normalized intensity and the corresponding reflectance value. The theoretical reflectance ratio, computed using the Radiative Transfer Equation (RTE) with an initial guess of the sample’s optical coefficients, allowed us to determine optimal correction factors. We matched the intermediary results to the theoretical reflectance ratio using a least-square fit. These procedures are crucial for the accurate computation of the subsequent 2D optical coefficients.

#### 2D nonlinear fitting in 3D-mDOI

We obtained a pair of parameters $\left(\mu_{a},\mu_{s}\right)$ for each pixel region utilizing the relationship between the reflectance R and the source-detector distance ρ described in the semi-infinite slab RTE (Figure S1). These parameters were computed using the Levenberg–Marquardt algorithm. Leveraging the quasi-heterogeneous assumption for the neighboring pixels, the tissue properties are considered uniform in a small neighboring region. The optical coefficients of the center pixel were computed with multiple (R,ρ) pairs from neighboring pixels in a cross section. In particular, the computation of the optical coefficients of a given center pixel was performed by separately utilizing (R, ρ) pairs from neighboring pixels arrayed in both horizontal and vertical configurations. To assure both consistency and accuracy in this process, we took the average of these independently derived results when finalizing the optical coefficients attributed to the same center pixel. A map of the 2D optical coefficients for each illumination patch was later generated by assembling these optical parameters pixel-by-pixel.

#### 3D reconstruction in 3D-mDOI

The structured illumination in multisite image acquisition platform was designed to match the 3D mapping of photon distribution for source-detector pairs in hybrid mathematical model. We isolated each light source illumination point within an illumination pattern to guarantee that each pixel value on the camera detector was influenced by only a single illumination point. In the mathematical model, each illumination point acted as a light source, and each camera pixel was viewed as an individual detector. Consequently, the intensity value for each source-detector pair is readily accessible, providing essential input for subsequent 2D optical coefficient calculations and the 3D photon distribution mapping. To ensure coverage of the entire sample region within the 3D coefficients matrix for each illumination source, we systematically shifted the light source illumination points based on a pre-designed scan pattern and reiterated the computation processes accordingly. (Figure 1D). This approach minimized nonuniformities in tissue reconstruction by ensuring multi-samplings of photon migrations from various directions.

The back-projection of the 2D optical coefficient to its associated photon’s 3D spatial distribution allowed us to achieve a 3D reconstruction. The most appropriate 3D photon distribution is chosen for each source-detector pair based on the source-detector distance ρ. This distribution maps the relevant optical coefficient to the 3D voxels that photons are more likely to traverse given the constraint of ρ (Figure 1C). It’s worth noting that each voxel in the reconstructed 3D matrix can have overlapping contributions from multiple 3D photon distributions originating from different source-detector pairs. To address this, we integrated each voxel from all 3D photon distributions contributing to it in two phases. First, we computed the 3D coefficients matrix for each illumination point and normalized it using a precomputed summation of all 3D photon distributions. We followed this by realigning these partial 3D coefficients matrices to their original positions within the captured image. A weighted average was applied to merge all the partial outcomes, resulting in a consolidated 3D reconstructed matrix for further calibration.

#### Uniformity calibration

Uniformity calibration, the final stage in 3D-mDOI, was designed to produce a calibration matrix and refined the reconstructed 3D coefficients matrix. Two distinct methods were available for this purpose, each with its own experimental and computational considerations. The first involved using a featureless, uniform phantom subjected to diverse illumination patterns. Although this technique provided a more consistent calibration matrix for subsequent reconstructions, it required both the presence of a uniform phantom and additional experimental steps. The alternative strategy was based on the idea that the majority of the phantom’s volume was concentrated in its base region rather than features. Through random sampling and averaging of reconstructed 3D patches, a representative 3D coefficients matrix for the base was created and utilized as the calibration matrix. While this method was more efficient for assessing samples that aligned with the given assumption, it might have affected accuracy. Regardless of the method selected, the resulting calibration matrix was used to correct each of the partial 3D reconstructions from experimental data, effectively minimizing grid artifacts and systematic noise (Figure 3D) and thereby accentuating the 3D optical coefficients of the features within the phantom.

#### Computation of region of analysis

We apply 3D-mDOI across varied analysis regions, aiming to achieve an enhanced and more accurate estimation of feature depth. According to the geometry of the photon distribution, the depth at the center of the 3D photon distribution extends to approximately half of the distance between light source and detector. The side length of the ROA area (Figure 4B) is restricted to four times that of the reconstructed depth of the feature. Given the physical restriction associated with the shape of the photon distribution, a 4mm-by-4mm ROA area, centered on the illumination point, enables a 1mm depth reconstruction. Consequently, the selection of the ROA for each illumination point is theoretically linked to the better quality of the depth estimation.

#### Finite Element Method reconstruction

The Finite Element Method (FEM) is commonly used to reconstruct steady-state diffuse optical imaging (DOI) by solving the light transport equation within tissue.^66^^,^^70^^,^^75^ FEM uses a forward model to predict the distribution of light fluence based on known optical properties and then solving an inverse problem to reconstruct these internal optical properties from boundary light fluence data. The model is iteratively adjusted to minimize the difference between measured and predicted data.

We utilized the open-source FEM MATLAB package^66^ and modified the input to fit OPTIMAP settings. Initially, we created the initial grid of voxels associated with the sample and assigned the locations of the light sources and detectors on the surface of the sample grid based on the simulation or experimental setup. Light sources and detectors were then linked together, and measurements were assigned according to these source-detector pairings. We then performed the inverse reconstruction in the form of standard steady-state DOI, with FEM optimizing the estimation of the optical coefficients for the entire sample mesh.

It is important to note that the input measurements used in this FEM analysis is the low SNR reflectance data collected by the low-cost OPTIMAP. Though this reflectance data do not align with the steady-state fluence used by FEM in other applications, it serves as our initial benchmark to evaluate the performance of the 3D-mDOI.

The entire sample grid was subdivided into smaller portions. Each subdivision was reconstructed independently. This was necessary due to the high RAM memory usage in FEM. Our OPTIMAP utilizes structured illumination, resulting in a large number of light sources and detectors pairs. This highly multiplexed approach leads to a large FEM matrix inversion size, exceeding 10 GB, making it impractical to reconstruct the entire sample all at once (Figure 3).

From the results of both simulated and physical phantoms, FEM presents reduced performance compared to 3D-mDOI, as FEM is not specifically designed to work within the constraints and capabilities of the OPTIMAP system.

#### Quantitative measurement for the synthetic phantoms

The normalization of reconstructed results from both 3D-mDOI and FEM is paramount to ensure consistent and unbiased quantitative measurements when comparing intensity-based distances and overall image quality against a ground truth. To facilitate an impartial comparison across various test scenarios, the reconstructions were scaled to a [0,1] range, while the ground truth was rendered into a binary function: the feature area was designated a value of 1 and the background, a value of 0. Employing these standardized outputs, several metrics were computed: Root-Mean-Square Error, which quantifies the deviation between a model’s reconstructions and actual simulated ground truth; the Bhattacharyya distance, a measure of the likeness between two histgram’s distributions; image contrast, which evaluates the disparity in optical coefficients that makes features distinguishable from base region; and the Multi-scale structural similarity index, evaluating image similarity aligned to the human visual perception.

The meticulous segmentation process plays a crucial role in ensuring an accurate and consistent assessment of segmentation errors between 3D-mDOI and FEM. To embark on this, manual segmentation of features was carried out from the reconstructions based on specific guidelines. Primarily, if a feature was perceptible from the surface, its shape in the initial reconstruction layer was expected to correspond to that of the ground truth. This stipulation draws from the understanding that an image modality other than the ground truth can provide insights into the feature’s surface structure, thereby setting a standard for manual segmentation. Furthermore, any chosen feature should be clearly distinguishable within the phantom, and its total volume should not surpass half of the phantom’s entirety. This not only sets a boundary for the reconstructed volume of the feature but also highlights the significance of establishing an appropriate scale for the multisite image acquisition platform. A feature should be ensconced with ample volume on all sides, ensuring that the 3D photon distribution effectively engulfs the feature from every direction, thereby facilitating a superior quality reconstruction. Following this, the manually segmented features were transformed into a binary format, akin to the processing of the ground truth. This binary representation sets the stage for calculating metrics such as the depth of the reconstructed feature, segmentation specificity, sensitivity, and the Dice coefficient, which gauges the congruence between segmented features from reconstructions and the ground truth.

##### Root-Mean-Square Error (RMSE)

RMSE is a frequently used metric to quantify the difference between a set of predicted values and the actual values they are meant to predict. $x_{reconst.}$ and $x_{gt}$ represent the pixel value of the reconstruction and ground truth, respectively. N is the total pixel number. RMSE measures the average of the pixel-wise error.

(Equation 2) $$RMSE=\sqrt{\frac{\sum_{1}^{N}{\left(x_{reconst.}-x_{gt}\right)}^{2}}{N}}$$

##### Bhattacharyya Distance

The Bhattacharyya distance measures the similarity between two probability distributions, especially for classes having similar mean values but different standard deviations. We computed the normalized histograms of the reconstruction and ground truth and the Bhattacharyya distance^76^ with respect to the two histograms was calculated. The Bhattacharyya distance of two histograms p and q over the same intensity range X is defined as the following.

(Equation 3) $$D_{B}\left(p,q\right)=-\ln\;\left(\sum_{x\epsilon X}\sqrt{p\left(x\right)q\left(x\right)}\right)$$

There is no ideal range for the Bhattacharyya distance. Generally, the lower the value, the higher is the similarity of the histograms.

##### Image contrast

While there are numerous methods to quantify the image contrast of a feature, for our study, we adopted a strategy that uses percentage contrast, calculated based on the disparity between the highest and lowest intensity values present in the reconstruction. This choice stemmed from an observation: the optical coefficients of feature reconstructions in 3D-mDOI exhibited a variance (Figure 3C), an artifact attributed to the illumination patterns. As a result, the distribution of optical coefficients for both features and the background closely followed a normal distribution in the reconstruction’s histogram. Recognizing this, we refrained from merely contrasting the peak intensity value against the lowest. Aiming for a more reliable and representative metric, we utilized a customized approach: the average of the top 20% intensities ($I_{Feat.}$) for the feature and the average of the bottom 20% intensities ($I_{B}$) for the background noise.

(Equation 4) $$Contrast=\frac{I_{Feat.}-I_{B}}{I_{B}}$$

##### Feature’s reconstructed depth

The depth of the reconstructed feature was determined from the binary function obtained from the segmented results. Given that the synthetic features were shaped as half circles, we utilized the deepest 1% of pixel depths within the feature for an average, providing a robust estimation of its size. To validate this depth, the segmented feature matrix was visualized for verification. Notably, each computed depth was based solely on a single manually segmented feature. Thus, the resulting depth could vary with changes in the segmentation.

##### Specificity, sensitivity and dice coefficient

The specificity, sensitivity, and Dice coefficient are commonly used to quantify the performance of segmentation. Specificity refers to the true negative prevalence for a feature, while the sensitivity corresponds to the true positive rate for a feature.

(Equation 5) $$Specificity=\frac{V_{gt\_bg}\cap V_{reconst\_bg}}{V_{gt\_bg}}$$

(Equation 6) $$Sensitivity=\frac{V_{gt\_feat}\cap V_{reconst\_feat}}{V_{gt\_feat}}$$

Where the $V_{gt\_bg}$, $V_{reconst\_bg}$ , $V_{gt\_feat}$ ,and $V_{reconst\_feat}$ denote the volume of the background in the ground truth condition, the volume of the background in reconstructed phantom, the volume of the feature in the ground truth and the volume of the feature in reconstructed phantom, respectively. The Dice coefficient jointly gauges the similarity of two samples.

(Equation 7) $$Dice\;coefficient=\frac{{2*\;V}_{gt\_feat}\cap V_{reconst\_feat}}{V_{gt\_feat}+V_{reconst\_feat}}$$

The reconstruction quality is better when all these three parameters are closer to 1.

##### Multi-scale structural similarity (MSSSIM)

The Structural Similarity Index (SSIM) was widely used to evaluate the quality of images, particularly in comparing an original to its degraded counterpart. This metric encompassed three primary attributes: luminance, which assessed the brightness; contrast, which highlighted variations in luminance or color, making distinctions in an image; and structure, which focused on inherent patterns and textures, providing the image its unique character. These elements collectively influence the viewer’s perception and interpretation of an image.

In our study, we observed that the shape of the 3D photon distribution, effectively acting as the point spread function of the system, caused the 3D-mDOI segmentation to cover a more expansive area than what was seen in the ground truth. This discrepancy led to a marked reduction in SSIM scores. To address this challenge, we turned to the Multi-scale SSIM (MSSSIM), a refined version of SSIM. MSSSIM introduced multiple levels of sub-sampling, allowing for a better representation of how the human brain perceives image quality.^77^ Adapting techniques from literature,^67^ we applied MSSSIM to each Z slice, comparing the ground truth with the reconstructed phantom. The averaged MSSSIM values over the z slices were presented in Table 1, showcasing our dedication to producing reconstructions that were not only precise but also visually authentic.

Consider two signals x and y arising from the same 3days coordinate of the ground truth volume and of a 3D reconstruction, in this work from 3D-mDOI or FEM. Let $\mu_{x}$, $\mu_{y}$ represent the mean of x, y respectively and, ${\sigma_{x}}^{2}$ , ${\sigma_{y}}^{2}$ represent the variance of x, y respectively . $\sigma_{xy}$ denotes the covariance between x and y. MSSSIM is fundamentally based on the comparison of measures of luminance l(x,y), contrast c(x,y) and structure s(x,y):

(Equation 8) $$MSSSIM\left(x,y\right)={\left[l_{M}\left(x,y\right)\right]}^{\alpha M}\cdot \prod_{j=1}^{M}{\left[c_{j}\left(x,y\right)\right]}^{\beta_{j}}{\left[s_{j}\left(x,y\right)\right]}^{\gamma_{j}}$$

Where $l\left(x,y\right)=\frac{2\mu_{x}\mu_{y}+C1}{{\mu_{x}}^{2}+{\mu_{y}}^{2}+C1}$ , $c\left(x,y\right)=\frac{2\sigma_{x}\sigma_{y}+C2}{{\sigma_{x}}^{2}+{\sigma_{y}}^{2}+C2}$ and $s\left(x,y\right)=\frac{\sigma_{xy}+C3}{\sigma_{x}\sigma_{y}+C3}$ are formulated based on the luminance estimation $\mu_{x}$, luminance contrast ${\sigma_{x}}^{2}$ , indication of structural similarity $\sigma_{xy}$ and three small constants $C_{1}=6.5$, $C_{2}=58.5$, $C_{3}=29.2$. α, β*,* γ are parameters that define the relative importance of the luminance, contrast and structure. In this work, we assigned a value of 1 to the relative importance parameters: $\alpha_{M}$, together with $\beta_{j}$*,* $\gamma_{j}$ at all scales. We compare contrast and structure between x and y at different scale j from 1 to M. For the luminance, we consider only the similarity at scale M.

### Quantification and statistical analysis

#### Quantitative measurement for the synthetic phantoms

The detailed analysis method is described in the STAR Methods section and the legend of Table 1. The quantitative measurement is implemented using Python 3.6.

#### Boxplots of relative optical coefficients ($\mu_{a}$, $\mu_{s}$) ratio of 3D-mDOI and FEM reconstruction for physical phantom features

The detailed analysis method is described in the legend of Figure 3 and implemented using Python 3.6.

#### Depth-dependent variance in 3D-mDOI recovered optical parameters

The detailed analysis method is described in the legend of Figure S4 and implemented using Python 3.6.

#### Estimation of the system imaging depth

The detailed analysis method is described in the legend of Figure S5 and implemented using MATLAB 2017b.

#### Stability analysis of diffused data captured by OPTIMAP

The detailed analysis method is described in the legend of Figure S9 and implemented using Python 3.6.

## Supplemental information

Document S1. Figures S1–S10, Table S1, Notes S1–S6, Method S1
