# Supplementary material for: Rapid diffused optical imaging for accurate 3D estimation of subcutaneous tissue features
Source: iScience. 2025 Jan 23;28(2):111818. doi: 10.1016/j.isci.2025.111818 (PMC11847144; doi:10.1016/j.isci.2025.111818)
Supplement: Document S1. Figures S1–S10, Table S1, Notes S1–S6, Method S1 [file mmc1.pdf]

iScience, Volume *28*

Supplemental information

Rapid diffused optical imaging for accurate 3D estimation of subcutaneous tissue features

Shanshan Cai, John Mai, Winn Hong, Scott E. Fraser, and Francesco Cutrale

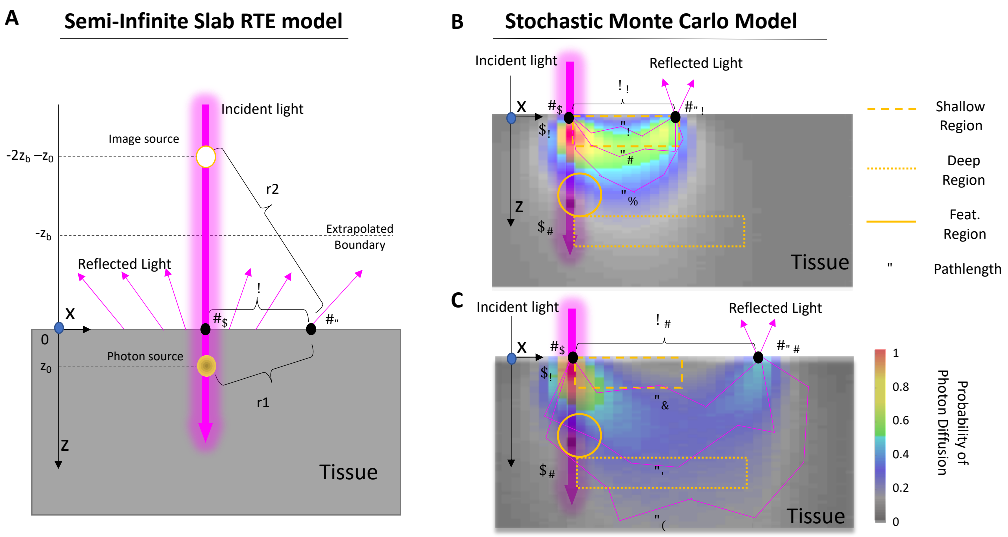

Fig.S1. Theoretical overview for two models of diffuse image formation, Related to Figure 1. A Beam of incident light (magenta beam) shines normally to the tissue surface at the location X_s. The diffused reflectance is detected at position X_d which is at a distance ρ from the source position X_s. In the (**A**) radiation transfer equation (RTE) model, the tissue is considered a semi-infinite slab. There exist two virtual isotropic light sources (e.g., the Image source, Photon source) that generate an equal photon diffusion with the tissue, as does the real incident light. The extrapolated boundary is where the light flux is equal to zero, providing the boundary conditions to the RTE model. The distances between the detector X_d and two virtual light sources (r_1, r_2) are used for fitting the optical properties of tissue. (**B**) Stochastic Monte Carlo simulation of the photons interacting with tissue at a distance ρ_1 , position X_{d1} and (**C**) a distance ρ_2, position X_{d2} accounting for locally different optical properties. The colors in the tissue phantom (**B** and **C**) denote the probability of the presence for a photon in each phantom voxel. The 3D tissue sample contains a subcutaneous blood vessel placed along the y axis (yellow circle), causing distortions in the shapes of the 3D photon diffusion maps. The maps for different distances ρ show the typical "banana" distribution, whose central depth is approximately half of the distance between the light source and the detector. A longer distance between the source and the detector leads to a deeper sampling depth. The distribution of photons with the smaller ρ (B) is more concentrated in a shallow region (yellow dashed rectangle) with more signals, while that with the larger ρ (C) spreads and samples deeper into the tissue model (yellow dotted rectangle) and presents increasingly reduced quantities of photons. Additional information is in Supplementary Note 1.

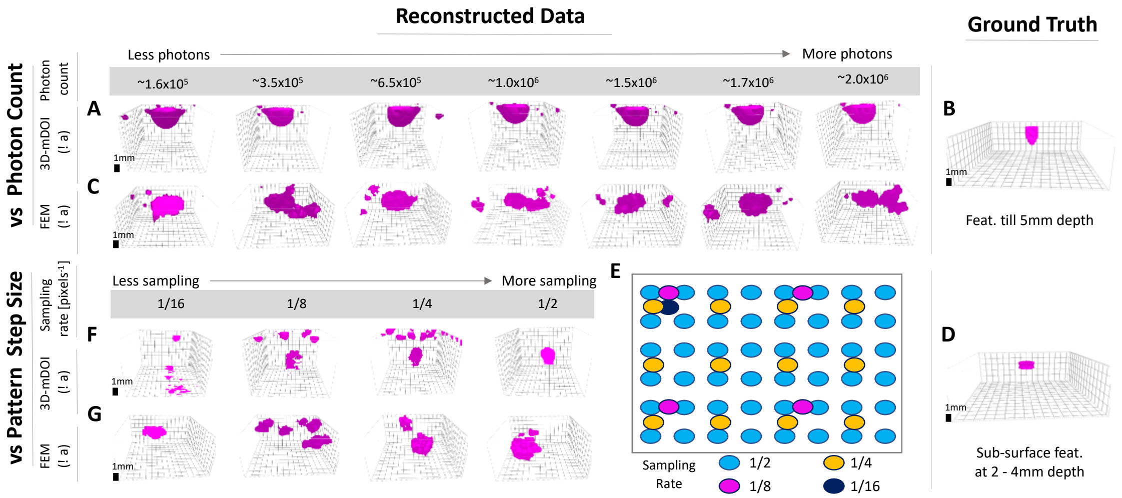

Fig.S2 The effect of photon counts and projected pattern size on the quality of the reconstructed volumes from 3D-mDOI and FEM simulations, using a numerical phantom, related to Figure 2. (**A**) 3D-mDOI reconstructed volume of a phantom with a feature extending 5 mm beneath the surface processed under different Signal-to-Noise Ratio (SNR) input conditions. (**B**) Reference ground truth for the 5mm deep feature. As the photon count increases, the quality of the 3D-mDOI reconstruction (A) generally remains constant. Higher photon counts translate to better SNR input and result in reduced noise at the surface. (**C**) Finite Element Method (FEM) results provide reduced subsurface details, even in a high photon count input scenario. (**D**) Phantom with sub-surface features from 2mm to 4mm to test the effects of different sampling rates (step size) of the sampling pattern, measured in $pixel^{-1}$. The lateral resolution of the phantom is 0.5 mm, and the projected sampling rates of $1/2$, $1/4$, $1/8$, and $1/16$ (**E**) correspond to illumination sampling steps of 1mm, 2mm, 4mm, and 8mm, respectively. For this test, reflectance is simulated from individual illumination points to preclude interference between illuminations. An increased sampling rate results in a denser distribution of illumination points on the sample, providing more source-detector pairs for an improved phantom voxel reconstruction. (**F**) 3D-mDOI reconstruction quality improves with an increase in sampling rate, with the tradeoff being a longer acquisition time. The 1/8 sampling rate results to an experimentally reasonable output with a faster acquisition time. (**G**) The reconstructions using the FEM approach have a low dependency on the illumination density.

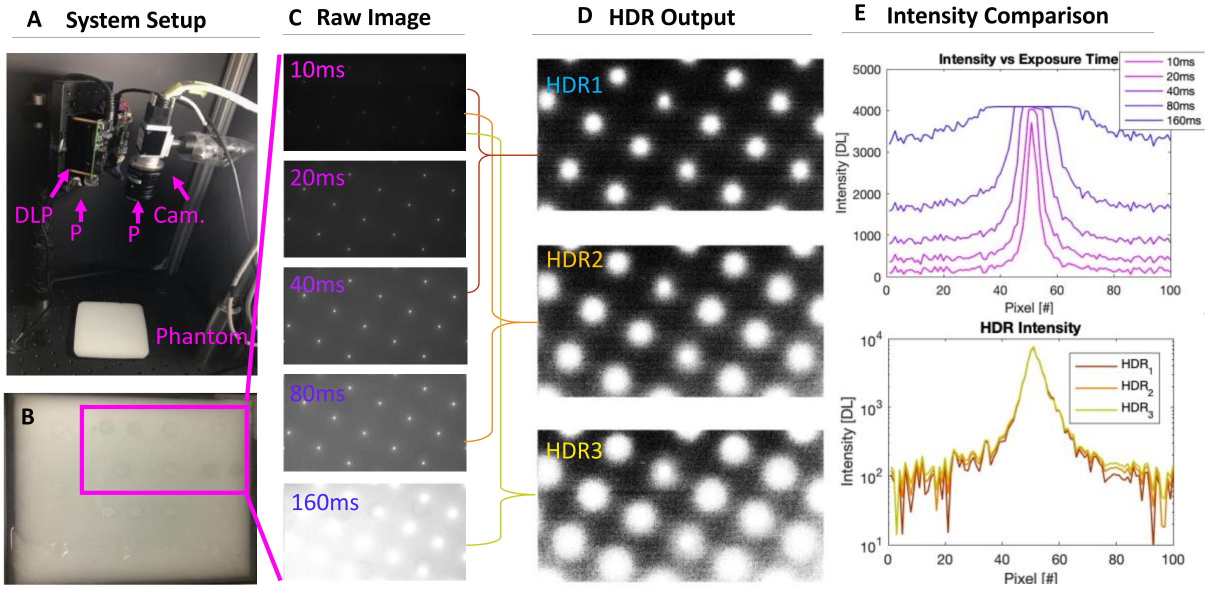

Fig.S3 3D-mDOI Acquisition Approach, related to Figure 3. A synchronized image acquisition platform is designed for capturing reflectance from tissue phantoms. (**A**) The Optical Properties Tissue Imaging Multiplexed Acquisition Platform (OPTIMAP) comprises a Digital Light Processor (DLP) for structured illumination and a high-speed 2 Megapixel CMOS camera with 12-bit pixel depth, both equipped with optical polarizers to considerably reduce specular reflectance. (**B**) A customized PDMS tissue phantom featuring insertions with varying optical properties is imaged using the platform in (A). We calculate 16-bit ultra-high dynamic range image to enhance the signal-to-noise ratio of the captured reflectance. Sets of raw images (**C**) are captured at varying exposure durations, with a constant illumination pattern. These raw images are then (**D**) processed to estimate three different 16 bit Ultra High Dynamic Range (UHDR1-to-3) images that utilize different subsets of the images in (C). The UHDR1-to-3 require a total acquisition time of 70, 150, 310 milliseconds respectively. (**E**) Top: A logarithmic scale plot of the intensity values at different exposure times, measured across one illuminating point at position (pixel = 50). Bottom: logarithmic scale plot of the HDR intensity value of the 3 HDR approaches shows an improved performance for UHDR2 and UHDR3 respect to UHDR1. In this work we utilized HDR2 owing to the reasonable performance with respect to the acquisition time. The UHDR in general shows a considerable improvement in the signal-to-noise ratio for distances of up to 45 pixels from the central light source.

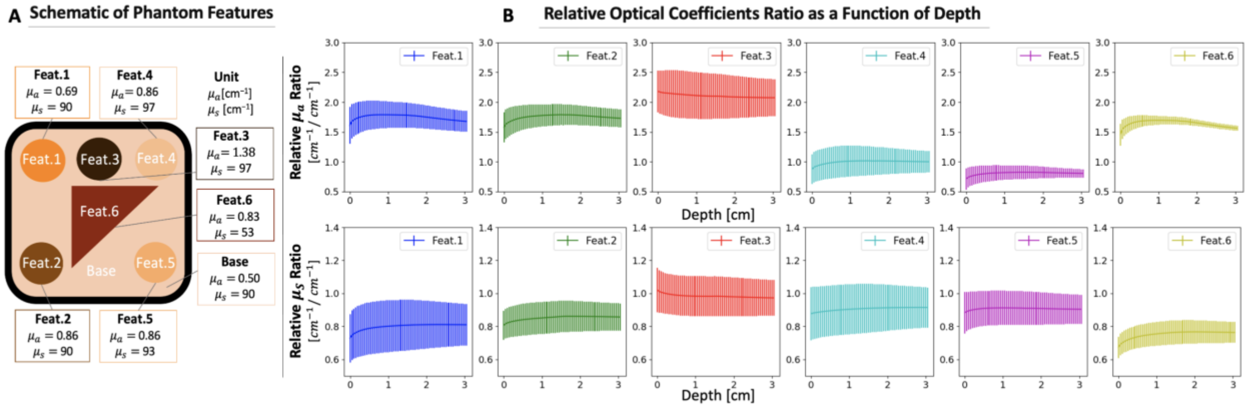

Fig. S4. Depth-Dependent Variance in 3D-mDOI recovered Optical Parameters, related to Figure 3. Plots representing the influence of depth on the calculated optical parameter values, emphasizing the effects of diminished photon sampling at deeper sections. Utilizing experimental measurements from a (**A**) physical phantom, we compute the ratio of each feature's optical property to the phantom's base for correcting the unevenness of the instrumental illumination. Ratios are reported for both relative (**B**) absorption (μ_a) and scattering coefficients (μ_s) over a sample of 10^5 voxels. Center line represents the average, error bars represent the standard deviation. While the shift in optical parameter value minimally affects post-processing operations, like feature segmentation, it compromises the precision of 3D-mDOI's depth estimation. The estimated correlation between the decay in these parameters and optical parameter pair values suggests the possibility of correction via advanced fitting via the introduction of a depth-incorporating lookup table.

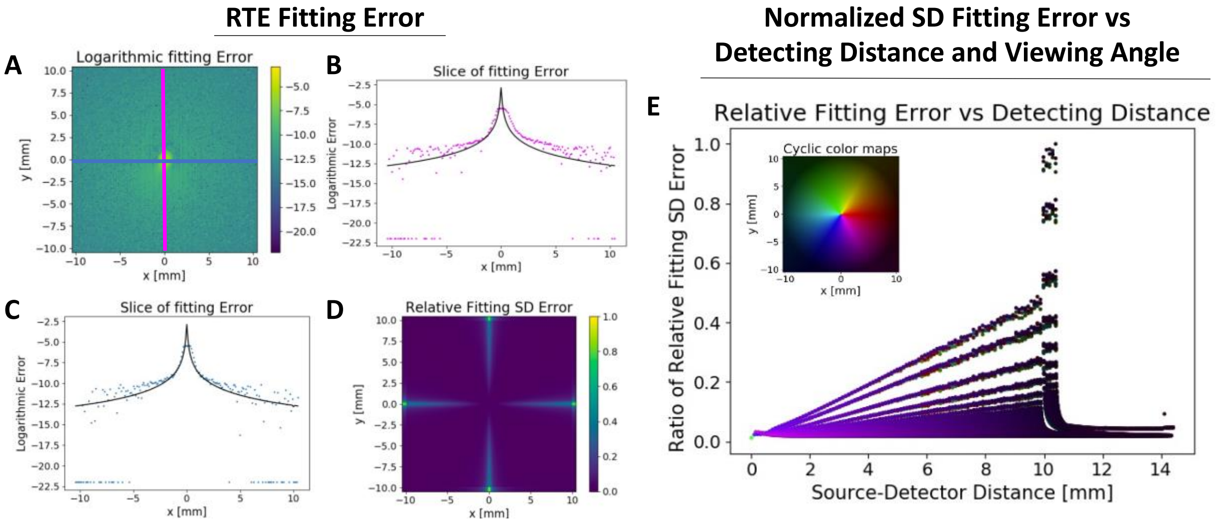

Fig. S5. Estimation of the system imaging depth using the fitting error from the Radiation transfer equation (RTE) , related to Figure 4. We analyze the fitting error for a uniform phantom with a single incident light beam centered at coordinates (0,0) to understand the physical limitations of 3D-mDOI reconstruction. A region of imaging containing 100*100 pixel is selected for the visualization and analysis. (**A**) 2D logarithmic plot of the absolute error between the experimentally acquired reflectance and the fitted curve, together with (**B**) its 1D profile along the Y axis and (**C**) along the X axis. The pixels close to the light source (A) have a larger fitting error due to the physical limitation of the RTE model. The pixels distant from the light source also have a larger fitting error caused by the lack of signal and diffusive information. (**D**) The 2D normalized standard deviation (SD) of the fitting error represents the stability of the nonlinear fitting and shows a cross-shaped artifact in 2D. This artifact is caused by the cross shape of the sampling pattern from a pixel-wise 2D nonlinear fitting of the sample's light parameters. (**E**) Plot of normalized fitting SD error with respect to the detection distance shows a linear correlation before the distance reaches 10mm. The error doubles with the distance ranges from 10mm to 11mm, where the SNR level degrades. The error drops close to zero when the distance is longer than 11mm because of the absence of a signal. The radial gradient descent color map (E, inset) encodes the angular information as colors and the distance from the center as decreasing brightness, showing that the fitting SD error is generally angle invariant.

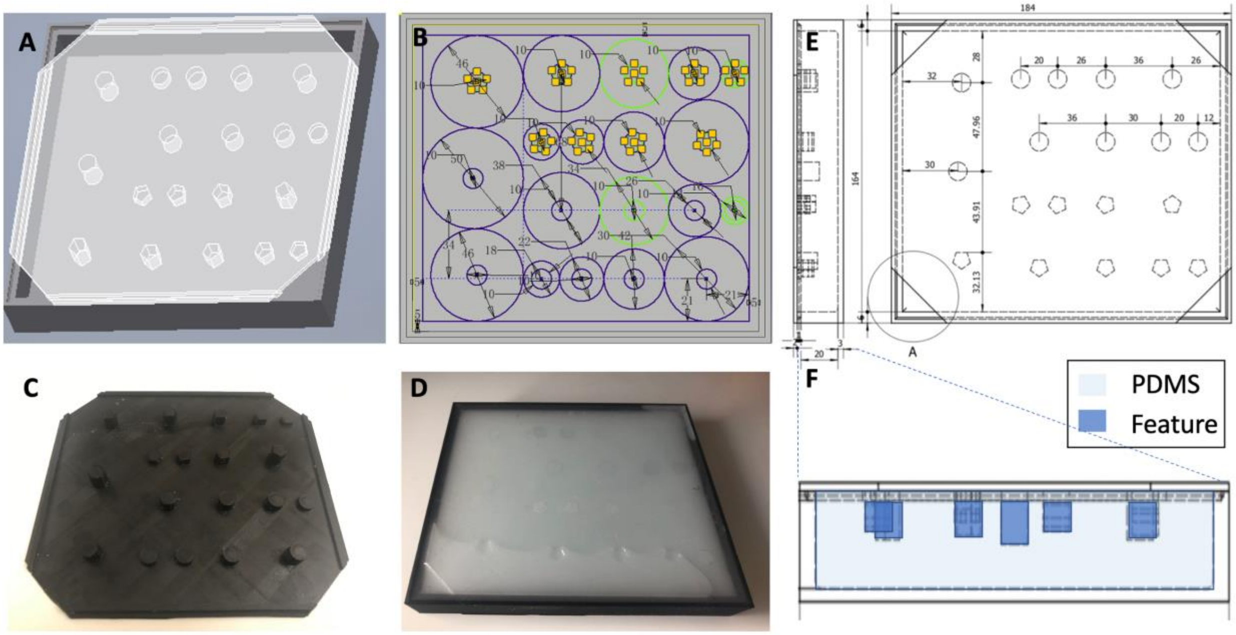

Fig. S6. 3D printed mold design and customized PDMS phantom assembly, related to Figure 3. (**A**) A rectangular container with a lid designed for generating PDMS phantoms with optical properties similar to human dermis tissues. (**B**) Schematic representation of the lid, both inner circles and pentagons depict features that have the same dimensions but differ in their protrusion depth. To ensure the full coverage of the characteristic 'banana' distribution during depth estimation, the region of analysis (Fig. S6) for a feature should extend at least four times its depth. By establishing concentric circles with a radius twice the depth of the features, we ensure these features are adequately spaced apart. The generation the customized PDMS tissue phantom requires two steps. First, the phantom-based area is cured by placing a (**C**) 3D-printed lid on the top of the container. Then, the feature areas left are filled with customized PDMS material, which possesses distinct optical properties (**D**). (**E**) Top and (**F**) side views of the PDMS phantom provide a comprehensive overview of its spatial characteristics.

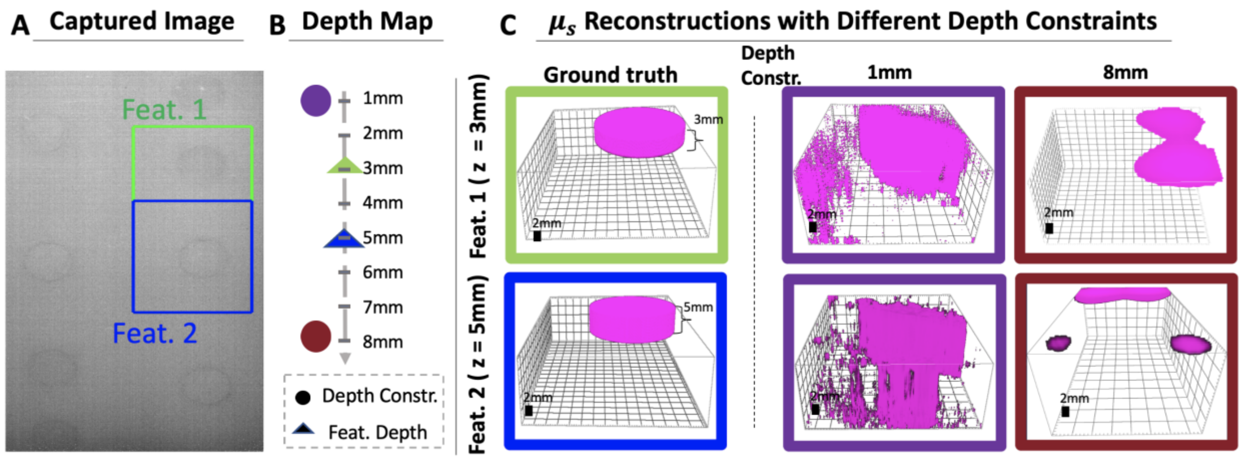

Fig. S7. Estimation of the system imaging depth reconstruction performance with variable depth constraints, related to Figure 4. The fidelity of reconstructing the features at various depths is analyzed under two extreme regions of analysis (ROA) constraints. Using experimental data acquired from (**A**) a physical tissue phantom featuring 3D-molded features at (**B**) depths of 3mm (B green triangle) and 5mm (B navy triangle) , we demonstrate the effects of overly constraining and excessively expanding ROA utilized for the reconstruction. The depth constraints are set at 1mm (B purple circle) and 8mm (B brown circle), under and oversampling depth, defining the minimum and maximum ROA thresholds for further experiment. These constraints are applied to the (**C**) reconstruction of features extending for 3mm and 5mm in depth (C, Ground Truth). Excessively restricted ROAs do not capture the crucial 'photon-banana' data, causing pronounced local noise in the reconstruction (C purple box), while oversized ROAs result in crosstalk and noise contamination, leading to a smoothed feature boundary (C brown box). These findings underline the significance of selecting an appropriate ROA depth to ensure accurate 3D reconstructions.

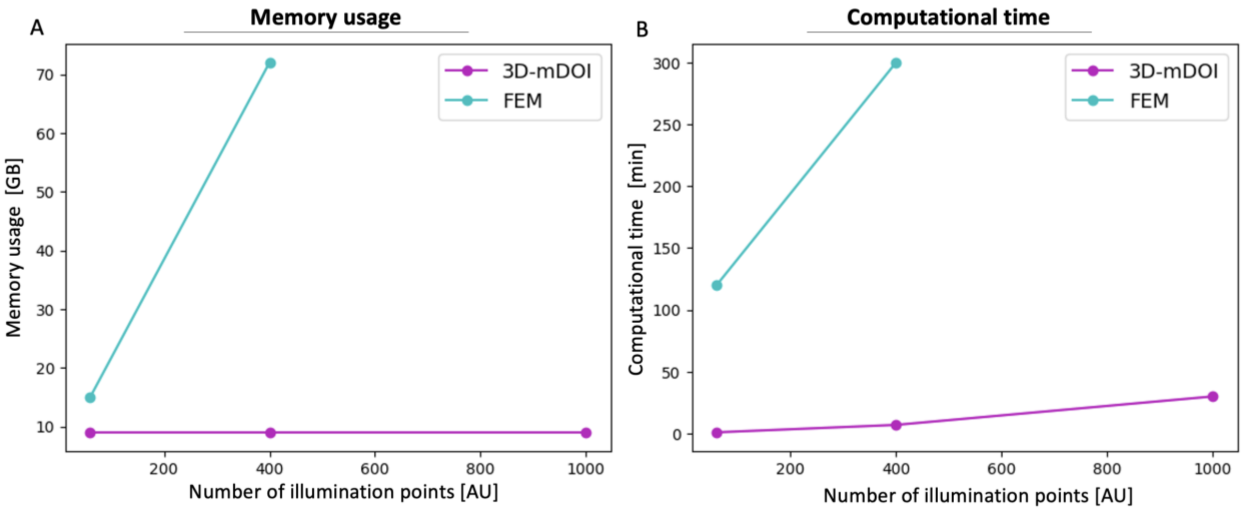

Fig. S8. Comparative Computational Efficiency – memory usage and time - of 3D-mDOI vs. FEM, related to Figure 3 and Table 1. We evaluate the computational efficiency of 3D-mDOI and Finite Elements Method (FEM) in reconstructing Feature 2 (main draft, Figure 4), utilizing 60, 400, and 1000 illumination points. (**A**) The memory usage of 3D-mDOI remains relatively steady at 9GB across all tests, irrespective of the number of illumination points. In contrast, FEM's memory usage scales with the illumination points, utilizing 15GB and 70GB for 60 and 400 points respectively. Notably, a computer with 128GB of memory failed to process the FEM reconstruction using 1000 illumination points. (**B**) Plot of the computational time shows an increase of 3 minutes for 3D-mDOI and 52 minutes for FEM for every additional 100 illumination points. It requires 3D-mDOI just 7 minutes to process Feature 2, while FEM took 5 hours, underscoring 3D-mDOI's marked advantage in computational speed. This up to 60-fold speed enhancement makes 3D-mDOI apt for many medical applications.

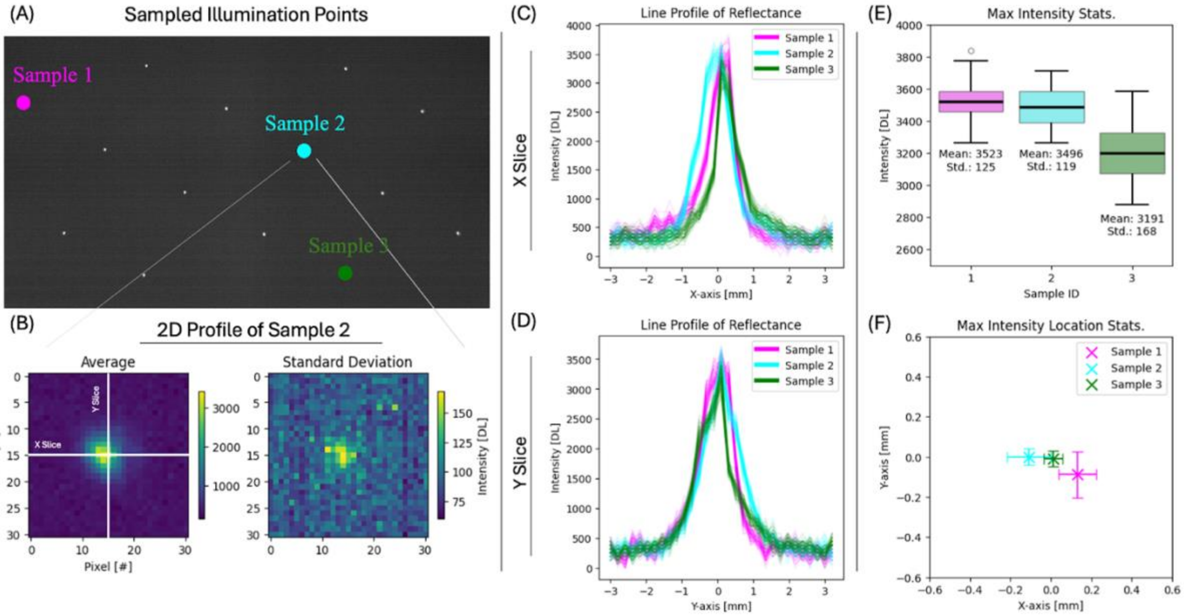

Fig. S9. Stability analysis of diffused data captured by OPTIMAP, related to Figure 1 and Figure 3. To assess the stability of diffused data captured by OPTIMAP, we capture 50 images of a physical phantom with a 20-millisecond exposure time. We analyze these images by selecting (**A**) three regions of interest (ROIs) of size 31x31 pixels (green, cyan, and magenta Sample 1 to 3), focusing on the intensity of reemitted light from different illumination points. (**B**) The average and standard deviation of the cyan dot across 50 images are computed, revealing a Standard Deviation of the intensity below 170 in the exponential decay area. We plot line profiles from three different sample illumination points for (**C**) X slice and (**D**) Y slice, from a total of 150 samplings. The profiles present minimal changes in light intensity for samples from the same illumination dot, while intensity profiles from different dots show slight shifts. To better estimate the stability, a box plot (**E**) is computed to show the distribution of maximum intensities for three sampled illumination points. The plot provides a statistical representation where the central box spans from the first quartile to the third quartile, bisected by a line representing the median for each sample illumination points. The whiskers extend to a maximum of 1.5 times the inter-quartile range, while any data points beyond these whiskers are denoted as flier points. (**F**) Scatter plot illustrating the spatial consistency and deviation in the locations of the maximum intensity among 50 images for the three sampled illumination points. Each point represents the location of maximum intensity for a specific sample, with the means marked by crosses (x) and the error bars representing the standard deviation in both X and Y directions. Both the stability of the maximum intensity in (**E**) and the consistency in the location of the maximum central point from multiple samples in (**F**) suggest that OPTIMAP has high stability (Supplementary Note 5).

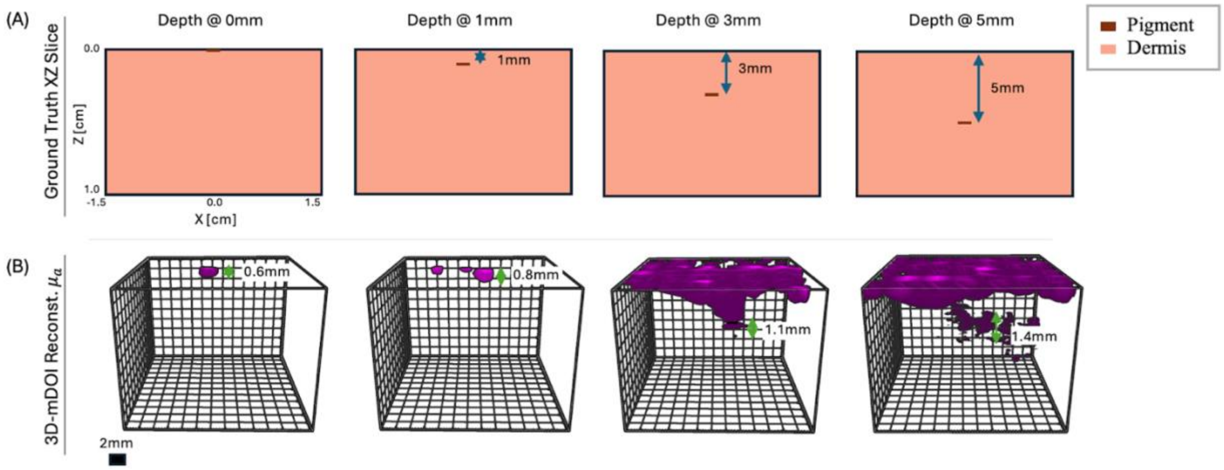

Fig. S10. Influence of sample depth on axial resolution in 3D-mDOI reconstruction, related to Figure 4. Schematic showing the placement of a disk-like pigment sample of 0.2mm thickness at various depths (0mm, 1mm, 3mm, and 5mm; **A** blue arrows) within dermis phantoms in the simulation. 3D-mDOI reconstructions of the pigment sample at the respective depths (**B** green arrow) , illustrating varying axial resolutions. The effective axial resolution of the sample becomes more diffused as the sample depth increases (Supplementary Note 6).

Table S1. Estimated optical parameters for two phantoms. Parameters are computed by measuring the concentration level of the TiO_2 and India Ink following protocols reported in literature (*14*).

Part 1: Estimated Optical parameters in phantom A

Feat.	1	2	3	4	5	6	b
μ_a [cm^{-1}]	0.69	0.86	1.38	0.86	0.86	0.83	0.5
μ_s [cm^{-1}]	90	90	97	97	93	53	90

Part 2: Estimated Optical parameters in phantom B

Feat.	Type1	Type2	b
μ_a [cm^{-1}]	0.5	0.2	0.2
μ_s [cm^{-1}]	100	200	100

Supplementary Note 1: Model for multisite Diffuse Optical Image Formation

Radiative Transfer Equation (RTE) stands as one of the central principles in the domain of Diffuse Optical Imaging, facilitating the depiction of radiation or light's pathway through a medium, such as biological tissue. This critical equation encompasses the phenomena of scattering and absorption that light encounters, as well as the exchange of radiative energy that occurs during these events. These interactions are characterized by three tissue properties that dictate the photon diffusion rate: the absorption coefficient μ_a, the scattering coefficient μ_s and the anisotropy parameter g. The reflectance ratio R is another important metric, demarcating the fraction of light reflected against that absorbed or penetrated the medium as a function of μ_a, μ_s, and g. Real-world measurement of this ratio entails the illumination of a test sample — such as a biological tissue — with a light source X_s, while a detector X_d quantifies the reflected light. By marrying the reflectance data with the RTE or its derived approximations, we can extract vital parameters like the absorption and scattering coefficients through optimization techniques. Effectively applying RTE pave the way for deeper understanding of sample's optical properties, enhancing our ability to study and analyze biological tissues.

By applying specific geometric and boundary conditions, analytical solutions to the RTE facilitate more rapid identification of the optical properties of test samples. To further alleviate the complex nature of the original RTE, a diffuse approximation method (*1*) is employed, an approach that assumes isotropic directionality of scattering events. A notable example is Farrell's model (*2*), which illustrates light interaction within a semi-infinite slab medium upon pencil beam light injection at X_s. The surface detector labeled X_d undertakes the role of measuring the reflectance, symbolized as $R(\rho)$, wherein ρ stands for the distance between the source of the light and the detector. The complexity of the incident light beam is reduced through the strategic use of two idealized light sources (Fig. S1): the photon source and the image source. The positions of these virtual sources are grounded in the medium's intrinsic characteristics, specifically its total interaction coefficient, denoted as $\mu_t{}'$. The location of the photon source is defined at a depth z_0 with $z_0 = \frac{1}{\mu_t{}'}$, while the image source is situated at a depth $-(2z_\mathrm{b} + z_0)$, where z_b represents the extrapolated boundary with zero photon diffusion. The distance between detector to two virtual sources are computed as $r_1 = [(z_d - z_0)^2 + \rho^2]^{\frac{1}{2}})$ and $r_2 = [(z_d + z_0 + 2z_b)^2 + \rho^2]^{\frac{1}{2}}$.

With the previously outlined assumptions, it becomes possible to model the reflectance $R(\rho)$, using a form b exponential decay (Eq.1). The effective attenuation coefficient $\mu_{eff} = [3\mu_a(\mu_a + \mu_s(1-g))]^{1/2}$, the transport albedo $a' = \mu_s(1-g)/(\mu_a + \mu_s(1-g))$, and total interaction coefficient $\mu_t{}' = \mu_a + \mu_s(1-g)$ are expressed in terms of μ_a, μ_s and g. This equation provides an approximate solution for reflectance as a function of distance from the source, based on the optical properties of the medium and the boundary conditions. Utilizing a least square fitting approach to align the experimental and theoretical reflectance data compute by Eq.1 further empowers a robust estimation of medium's optical properties.

$$R(\rho) = \frac{a'}{4\pi}[\frac{1}{\mu_t{}'}\left(\mu_{eff} + \frac{1}{r_1}\right)\frac{e^{-\mu_{eff}r_1}}{{r_1}^2} + (\frac{1}{\mu_t{}'} + 2z_b)(\mu_{eff} + \frac{1}{r_2})\frac{e^{-\mu_{eff}r_2}}{{r_2}^2}] \quad (1)$$

Monte Carlo simulations serve as another pivotal tool in demonstrating tissue photon migration, creating a discretized volume of the tissue, and then solving for photon-tissue interactions stochastically in each voxel. Each launching photon undergoes scattering, absorption, and directional changes until their energy is depleted. The interactions are computed repeatedly to obtain statistically significant 2D reflectance at the tissue-air boundary. This type of simulation accounts for the diversity and structural complexity of depth-related optical diffusion in tissues. Although generally more computationally demanding compared to the RTE model, it can provide a statistical representation of diffuse reflectance for a heterogeneous 3D medium (Fig. S1, B and C). A noteworthy phenomenon here is the formation of a 3D spatial probability

density function characterized by a photon-banana shape (*3*) as photons travel between a light source and a detector separated by a distance ρ. The central depth of this distribution correlates to half the distance ρ (Fig. S1B). While expanding the source-detector interval facilitates deeper and voluminous tissue samplings, it inversely affects the photon detection rate, diminishing exponentially with growing ρ (Fig. S1C).

Within the theoretics framework of scattering media imaging(*4*), the steady-state radiance, accounting for the energy flow of that light from a certain point over an area and within a given angle, represented as $S_m(X_s, X_d)$ (Eq. 2), at point X_d is determined by three primary elements: the light source, the detector, and photon-medium interactions. $W(X_s)$ denotes the emittance function of the light source, providing insight into how photons are emitted from the source. $W(X_d)$ refers to the point spread function of the detector, illustrating how the detector perceives or collects photons. In an ideal steady-state imaging situation, both $W(X_s)$ and $W(X_d)$ are typically constant, not varying across experiments. $W(\tau)$ provides the probability of selecting photons with a trajectory length represented as τ. Ideally, for every value of τ, $W(\tau)$ is 1, suggesting no further selection based on trajectory length in our experiment. $\mathcal{T}_m(X_s, X_d, \tau)$ is the remaining radiance reaching the detector after photons have interacted with the medium along their respective trajectories. The term can be computed by Monte Carlo simulation with known medium optical properties with constraints set for fixed values of (X_s, X_d, τ).

$$S_m(X_s, X_d) = \int_0^\infty W(X_s) W(X_d) W(\tau) \mathcal{T}_m(X_s, X_d, \tau) d\tau \qquad (2)$$

Given the underlying assumption of photon independence in relation to both the source and detector (*5*), and considering the characteristics of our experimental setup, we simply $S_m(X_s, X_d)$ with experimental correction $K(X_s, X_d)$ and $\mathcal{T}_m(X_s, X_d)$as Eq.3. In the situations we examine, the CMOS camera captures reemitted steady-state radiance where the incident light projected by the DMD interacts with testing sample. $K(X_s, X_d)$ is an experimental expression of acquisition parameters $W(X_s)W(X_d)W(\tau)$. The factor $A(X_s)$ accounts for unevenness in the illumination, leading to vignetting. $A(X_s)$ also corrects for the ambient background lighting which is assumed to be constant during the experiment. $C(X_d)$ is a calibration constant which accounts for camera detector noise, such as hot pixels, camera readout and background noise.

In contact-free imaging, $W(\tau)$ in Eq. 2 varies due to photons leaking between neighboring detectors. The scattering nature of photons may cause them to be emitted from tissue at angles not perpendicular to the surface. Consequently, there is a higher chance of them being detected by a neighboring detector rather than the one directly aligned with them, particularly in contact-free imaging as compared to contact imaging. We remove $W(\tau)$ in Eq. 2 and introduce a corrected pattern $P(X_s, X_d)$ to K to simplify the model, compensating for the energy loss when light travels back from the medium surface to the detector. The integral expression $\int_0^\infty \mathcal{T}_m(X_s, X_d, \tau) d\tau$ can then be represented more succinctly as $\mathcal{T}_m(X_s, X_d)$, given that we account for all the photons reemitting at X_d.

$$S_m(X_s, X_d) = K(X_s, X_d)\, \mathcal{T}_m(X_s, X_d) \qquad (3)$$

Given

$$K(X_s, X_d) = A(X_s) * C(X_d) * P(X_s, X_d)$$

Building on the information provided earlier, the inverse problem of photon migration via Monte Carlo simulation can be addressed by determining the optimal inference of the medium's optical properties. This is achieved by using stochastic gradient descent to minimize the discrepancy between the theoretical and experimental radiance values. However, addressing the inverse problem of the Monte Carlo model

utilizing steady-state measurements poses two primary obstacles: computational time and result ambiguities. First, the μ_a and μ_s estimations are extremely computationally intensive and impractical for reconstruction of the optical properties for large, high-resolution volumes(*6*). Secondly, determining the attributes of non-uniform tissues remains undetermined, with the potential for multiple viable solutions (*4*). Given that 2D diffuse reflectance is recorded solely at the tissue surface, there arises a complication; photons traversing from the source to the detector (X_s, X_d) can undertake a virtually limitless array of paths, a complexity that magnifies in the presence of tissue heterogeneity.

We develop a hybrid mathematical model, merging the 2D analytical insights garnered from the RTE model with 3D photon diffusion maps, shaped through Monte Carlo simulations. Employing a digital mirror device (DMD) to manage the incident light, a 2-megapixel camera precisely captures the reflectance data, with each pixel functioning as a separate detector. Initially, we lean on the RTE model to yield a 2D depiction of surface-level optical properties. Despite RTE's restriction to homogeneous tissues, we hypothesize that a pixel subset in a small region represent a quasi-homogeneous structure. This perspective enables us to construct μ_a and μ_s maps indicative of the region's optical characteristics. Progressing from this 2D representations, we extend them into the 3D space, adhering to the precomputed 3D-photon diffusion distributions generated through Monte Carlo simulations.

In line with our quasi-homogeneous extension into 3D, we computed 3D photon diffusion lookup maps on a homogeneous standard phantom. These maps, prepared for various light source-detector distances, present spatial distributions for an estimated 50 million photons. When refining our mapping strategy, we made a key assumption: melanin level variations primarily affect the absorption coefficient, whereas the scattering coefficient largely dictates the photon's trajectory within the medium. Given our emphasis on melanoma, we model obvious change in absorption coefficient with minimal scattering coefficient variance (*7*) We employ a consistent lookup map for all tested samples, hypothesizing that potential deviations in this map, resulting from optical property shifts, are minor (*8*). Moreover, we are provisioned for these subtle variations to be rectified in later multi-calibration phases with joint math model.

To mathematically conjoin the two models, the reflectance in the semi-infinite slab RTE needs to be represented as reemitted radiance in the Monte Carlo model. The transformation between reflectance to radiance through Eq.4 is based on two of assumptions. One, the scattering coefficient in tissues typically overshadows the absorption coefficient, leading to a small absorption decrease of the incident radiance. Two, by ensuring adequate spacing between the light source and detectors, reemitted signals captured by nearby detectors primarily emit from a single light source. Hence, R(ρ), the reflectance ratio, mirrors the ratio of radiance reflected at X_d over the total from all detectors. Within the scope of our analysis, $S_m(X_s, X_d)$ is a direct product of $K(X_s, X_d)$ and $\mathcal{T}_m(X_s, X_d)$ (Eq.3). Recognizing that the Monte Carlo simulation naturally captures the 3D spatial distribution in its calculation of $\mathcal{T}_m(X_s, X_d)$, it is justifiable to transition the 2D optical properties from RTE into a 3D perspective, leveraging the 3D distribution deduced from Monte Carlo methods.

Eq.4 also contains $\gamma_m(\rho)$ which is a correction term necessary to bridge any inconsistency between the two mathematical models. Generally, the differences in the reflectance obtained via RTE or Monte Carlo simulations, under the same experimental conditions, is negligible. However, when ρ is small, the resulting radial distances are smaller than half of the mean free paths of light in the tissue. Thus, any mismatch between the two models result in systematic errors leading to an invalidation of the diffusion assumptions(*2, 9*). $\gamma_m(\rho)$ is originally determined from both the distance ρ and the optical properties of the medium. In our scenarios, we utilize a fixed γ_m for the inconsistencies with corresponding to medium properties. This rationale stems from our approach wherein every voxel undergoes multiple samplings, ensuring a thorough accounting for tissue heterogeneities. Regions prone to systematic errors due to sampling at smaller ρ values are inherently corrected by samples taken at greater distances (*10*). Consequently, the influence of $\gamma_m(\rho)$ on the inverse model is effectively averaged out.

$$R(\rho) \cong \gamma_m(\rho)\frac{S_m(X_s,X_d)}{\sum_{d\epsilon D} S_m(X_s,X_d)} = \gamma_m K(X_s,X_d)\frac{\mathcal{T}_m(X_s,X_d)}{\sum_{d\epsilon D} S_m(X_s,X_d)} \qquad (4)$$

Through the above analysis, the efficient reconstitution of the 3D optical coefficient matrix is realized by transposing the 2D optical coefficient information into the 3D space, syncing it with a pre-determined 3D photon distribution map. In our approach, every voxel undergoes oversampling via numerous source-detector pairs, guaranteeing a thorough consideration of photon interactions. This methodology minimizes inaccuracies stemming from inconsistencies in optical coefficients, often attributed to uncertainties in light travel over diverse distances.

Supplementary Note 2: 3D-mDOI experimental parameters exploration

The 3D-mDOI reconstruction's precision hinges on the signal-to-noise ratio (SNR) of the reflectance image, dictated by factors like acquisition parameters, light injection power, and sampling frequency from varied illumination sources (Fig. S2). Elevating the incident light's power, signified by an uptick in simulated photons for every illuminating point, is one approach to improve the SNR. When reconstructing the deeper regions of a sample, it's crucial to rely on reflectance data from source-detector pairs positioned further apart. Due to the energy loss or absorption faced by photons on longer trajectories, increasing the photon count is crucial for more reliable observations of such regions. Another quality booster is a heightened sampling frequency, which is inversely proportional to the step size between consecutive illumination points. The increment in sampling frequency ensures that an individual voxel receives input from a multitude of source-detector pairings, encompassing varied 3D-photon migration paths. However, a surge in SNR, while enhancing precision, also elongates the acquisition duration. Therefore, striking the right balance in acquisition parameters is key to the 3D-mDOI method's efficiency.

Employing a gradient of simulated photon counts, we carried out multiple reconstructions of a consistent 5 mm deep feature across a spectrum of SNRs (Fig. S2A). The 3D-mDOI volumetric reconstructions show a consistent estimate of the 3D shape of the feature, across a wide photon range from 1.6 - 20.0 x 10^5 photons, supporting the robustness of our approach. The position of the reconstructed feature matches the ground truth (Fig. S2B). The volume is predominantly free from distortions and anomalies, though some are observed at its surface. Contrarily, while the FEM accurately pinpoints the feature's position, its results exhibit notable distortions and increased anomalies (Fig. S2C). Much like the 3D-mDOI, the FEM reconstruction quality does not markedly improve with an increased photon count. These observations suggest the presence of a saturation point beyond which intensifying the light's strength has a diminishing impact on the final outcome's quality.

Continuing our exploration, we assess the effects of different sampling frequencies (Fig. S2E), ranging from 1/16 to 1/2 per pixel, on the reconstruction of a synthetic phantom with sub-surface features (Fig. S2D) between 2mm and 4mm in depth. In general, a higher sampling rate enhances reconstruction accuracy by addressing the undetermined regions of the geometry and reducing artifacts (Fig. S2F). At the sparsest sampling rate of 1/16 per pixel, 3D-mDOI struggles to accurately depict the sub-surface feature, primarily due to an inadequate number of source-detector pairs intersecting the central feature. When sampling rate increases to 1/8 and 1/4 per pixel, we observe accurate reconstruction of the feature's position, albeit with some geometrical distortions and surface artifacts. By the time the sampling rate reaches 1/2 per pixel, artifacts become negligible, resulting in a commendable reconstruction outcome. Contrastingly, the FEM methodology (Fig. S2G) deviated from this pattern. At the 1/16 rate, FEM computes a reconstruction closely resembling the target output but placed at a different location. While FEM reconstructions refine with narrower intervals, the outcomes frequently skew in geometry, position, and exaggerated artifacts. One could infer from these findings that this specific task is intrinsically arduous for FEM, implying that simple enhancements in sampling frequencies may not redeem FEM reconstructions for such test cases.

Supplementary Note 3: OPTIMAP experimental boundaries of performance

To understand the system limitations of 3D-mDOI's reconstruction depth, we compute the fitting error of Radiative Transfer Equation (RTE) (Fig. S5, A, B and C) and its standard deviation (SD) (Fig. S5, D and E) for a single light beam onto the uniform physical phantom. A higher fitting error indicates a poorer approximation of the phantom's optical properties, whereas a larger standard deviation points to the inconsistency or instability of the fitting. We assess the fitting error to determine the depth at which the reconstruction remains accurate. Similarly, by evaluating the standard deviation, we ascertain the depth limit where the reconstruction can be deemed reliable and trustworthy.

The fidelity of the fit is depicted through showcasing the logarithmic absolute fitting error (Fig. S5A) along with its x, y profiles (Fig. S5, B and C). We observe a diminishing performance as the source-detection distance approaches 5 mm. Theoretically, the central depth of 3D photon distributions is about half of the source-detector distance. This trend indicates a satisfactory fit up to a depth of approximately 2.5 mm. The normalized SD of this error (Fig. 5, D and E) offers insight into the reliability of the fit. Notably, the SD error exhibits a linear uptick until the source-detection distance hits 10 mm, at which point it doubles (Fig. S5E). This abrupt increment in error is attributed to the edge effect of our test phantom, resulting in an isotropic sampling discontinuity in 3D-mDOI. When the source-detection distance is longer than 10mm, undetectable reflectance signal for the pixels lead to the reduced SNR and a drop in the SD fitting error. Hence, a practical 10mm limit in the source-detector distance translates to an approximate reconstruction depth cap of 5 mm, with depths up to 2.5 mm being reconstructed optimally. These derived parameters are subsequently utilized to determine the optimal depth for enhanced reconstruction.

We discern a 2D pattern in the fitting error emerging from the central pixel extending along the x and y axis, a byproduct of the 2D nonlinear fitting procedure inherent in 3D-mDOI. When the light source distance is less than 0.5 times the mean free path of the photon, the pixels have larger absolute fitting error (Fig. S5A), an inherent mathematical error when solving Radiative Transfer Equation in this situation. In 3D-mDOI we use neighboring reflectance data to determine the properties of a central pixel. Due to the reliance on this central data, which has a higher system error, the resulting areas along the x and y axes stemming from this central pixel exhibit an enhanced relative fitting SD error. This spatial error pattern, emerging in the shape of a cross, can be expected given the Radiative Transfer Equation used. On a 2D map (Fig. S5D), the relative fitting SD error is distinctly displayed as a cross pattern. Apart from this, the fitting SD error maintains its consistency across different viewing angles, underscoring the isotropic nature of 3D-mDOI. The uniformity calibration is an astute solution to ease this system's inherent errors. By doing so, we capture and retain the cross artifact observed in the uniform sample (Main draft Fig. 1E). When assessing samples with optical properties that differ from the uniform sample, the pronounced cross pattern becomes less evident. This reduction in artifact visibility is a proof to the uniformity calibration's efficacy in representing the general trend of the RTE fitting error.

Supplementary Note 4: 3D-mDOI computational efficiency

Evaluating the computational memory efficiency of 3D-mDOI reveals significant advantages in both memory usage and processing speed during data analysis. Unlike the FEM, where memory consumption scales linearly with both the number of voxels and of light source-detector pairs, 3D-mDOI's memory requirements scale linearly only with the number of light source-detector pairs (Fig. S8). For example, FEM-based reconstruction of Feature 2 (Main draft Fig. 4), necessitates a substantial 70GB of virtual memory, while the comparable process in 3D-mDOI requires just 9GB (Fig. S8). The traditional FEM model can easily run into memory overflow, on a standard research workstation, when incorporating numerous light source and detector pairs due to the method's extensive memory requirements. In contrast, 3D-mDOI accommodates large sets of light source and detector pairs without sacrificing computational efficiency.

In terms of computational speed, 3D-mDOI's is marginally outpaced by FEM in the context of synthetic phantoms reconstructions. Specifically, a numerical phantom reconstruction (Main draft Fig. 2 and S2), is accomplished within 200 seconds utilizing an optimized FEM package, whereas 3D-mDOI requires approximately 300 seconds. This trend is inversed with physical phantoms: 3D-mDOI reconstructs Feature 2 (Main draft Fig. 4) in approximately 7 minutes, a stark contrast to FEM's 5-hour requirement (Fig. S8). This extended time is caused by the higher number of light source-detector pairs in physical phantom reconstruction, which increases the iterations needed for FEM's accurate fitting, prolonging its computational time. It should be further noted that 3D-mDOI offers potential for further optimization. Implementing a lookup table (*11*) and introducing parallel processing (*12*), could notably reduce its reconstruction time.

Supplementary Note 5: Sensitivity and stability analysis of OPTIMAP

Stability

To further examine the stability of the diffused data captured by OPTIMAP, we captured 50 images of the physical phantom with the same imaging settings of OPTIMAP, using an exposure time of 20 milliseconds (Fig. S9 Sample 1 to 3). With this raw data, we selected three regions of interest (ROIs) of size 31x31 pixels corresponding to an area of 6.2 by 6.2 mm, focusing on the intensity of the reemitted light from different illumination points spread out in the captured image (Fig. S9 A, green, cyan, and magenta dots).

The average and standard deviation of the 2D profile of sample 2 across 50 images are shown in Fig. S9 B. The plots indicate that variability is minimal in the area of exponential decay which is fitted to reconstruct light coefficients. The standard deviation in the central decay area is under 50, suggesting that the 2D diffuse profile of reemitted light captured by OPTIMAP is generally stable over time. We plotted the X slice and Y slice line profiles (Fig. S9 C and D) from a total of 150 samplings across three different illumination points. The line profiles for the same sample exhibit minimal changes in light intensity. The line profiles show a subtle shift between different samples, generally indicating illumination unevenness due to the projecting angle caused by the projector. This shift can be further mitigated by corrections such as flat-field correction and uniform calibration.

We also analyzed the stability of intensity values within each cluster, indicating the spread of the maximum intensities and locations from their respective means. We applied a box plot (Fig. S9 E) to display the distribution of maximum intensities for three sampled illumination points. The percentage of fluctuation over the center value for intensities is calculated by dividing the standard deviation by the mean and multiplying by 100. For samples 1, 2, and 3, the fluctuations are 3.5%, 3.4%, and 5.2%, respectively, with an average fluctuation of 4% in the maximum intensity of the dot. A scatter plot (Fig. S9 F) illustrates the spatial consistency and deviation in the locations of the maximum intensity among 50 images for the three sampled illumination points. Each point represents the location of maximum intensity for a specific sample, with the means marked by crosses (x) and the error bars representing the standard deviation in both X and Y directions. We can conclude that the location of the maximum intensity is generally within the -0.2 to +0.2 mm range, which is 6.5% of the entire range of the diffused pattern.

Based on these computed metrics, the stability of OPTIMAP is demonstrated by the low percentage fluctuation in intensity values and the tight clustering of maximum intensity locations. This indicates a high level of consistency and reliability in the performance of OPTIMAP across different sample locations and acquisition settings.

Ensuring the system stability remains a complex and open problem and with room for further improvement. Despite applying multi-stage calibrations to improve system stability after OPTIMAP data collection (Methods), illumination unevenness due to vignetting in the optical path still affects the performance of our reconstruction, particularly for μ_a extraction, for example in Features 4 and 5 (main draft, Fig. 3). Future work will further improve the stability of the acquisition process.

Sensitivity

Several factors could affect the sensitivity of the system, including the sensitivity of the detector and further enhancement through preprocessing methods. The CMOS camera used to collect the reflectance data plays a crucial role in determining the sensitivity level of the detector. The dark noise level of the CMOS camera is approximately 13.9 e^- (Basler acA2000-340km, Technical Specifications, Basler Website), indicating moderate performance in low-light conditions. An SNR of 39.7 dB suggests that our

camera can produce relatively clear images with good separation between signal and background noise, indicating good sensitivity.

We reported the acquisition approach of OPTIMAP and the profile of the raw data in Fig. S3, along with the methods used to boost the dynamic range of the OPTIMAP acquisition. We further enhance the system's sensitivity through several preprocessing methods (Methods). These methods include carefully designing the illumination pattern to ensure that the reemitted light from multiple illuminations does not overlap and employing multiple exposure times for the sample with the same setting, enabling a 16-bit ultra-high dynamic range (UHDR). The raw intensity captured by OPTIMAP undergoes preprocessing and is converted into reflectance data (Fig. S5). According to our analysis in Supplementary Note 3, 3D-mDOI is sensitive to diffused light from a maximum depth of 5 mm within the physical phantom.

Supplementary Note 6: Impact of feature depth on 3D-mDOI axial resolution

The estimated reconstruction axial resolution for the 3D-mDOI is influenced by multiple factors that affect the amount of information collected by the detector, including illumination intensity, optical properties of samples, feature depth, and the number of patterns. Here we demonstrate how feature depth affects axial resolution through a series of simulations (Fig. S10). We conducted a series of simulations with a disk-like pigment feature of 0.2 mm thickness. We placed the feature at depths of 0 mm, 1 mm, 3 mm, and 5 mm (Fig. S10 A, blue arrows) in the dermis phantoms and observed the corresponding changes in the 3D-mDOI reconstruction.

In our simulation, the 0.2 mm thickness of the pigment feature is utilized to match the reconstructed phantoms' voxel size of 0.5 x 0.5 x 0.2 mm. The voxel size is limited by the shapes of the 3D photon distributions and the difference in absorption/scattering coefficients between the measured features and their surroundings. A 0.2 mm axial voxel size balances the computation size and the ability to distinguish millimeter-range features for potential use in identifying melanoma stages.

3D-mDOI reconstructs distinguishable features in all four subsurface cases up to 5 mm deep in the simulated dermis phantom(Fig. S10 B). As the feature depth increases, the reconstructed phantom presents more broadening of the structures, with additional artifacts appearing on the surface and surrounding area of the reconstructed features. The reconstructed feature at a depth of 1 mm is too broad to be distinguished as a distinct subsurface feature. At a depth of 3 mm, the reconstructed feature begins to show a trend separating from the artifacts on the phantom surface, and a subsurface reconstruction is observed at a depth of 5 mm. We measured the thickness of the subsurface feature (Fig. S10 B, green arrows), disregarding surrounding artifacts and noise, to estimate the reconstruction axial resolution.

The results suggest that the estimated reconstruction axial resolution decreases linearly with depth, exhibiting a broadening of the subsurface features. This phenomenon is a consequence of how the photon migration probability distribution shape changes with depth, resulting in increasingly larger "banana" distributions as light samples deeper locations, which compromises the axial resolution for the reconstruction.

The purpose of this experiment was to understand the effect of depth on the reconstruction's axial resolution. In typical dermatology application, subsurface features have limited relevance for melanoma diagnosis. Melanoma skin cancer commonly onsets in melanocytes located in the basal layer of the epidermis, the outermost skin layer (*13*). Therefore, the deteriorated reconstruction of subsurface features generally would not impact melanoma diagnosis. We report the performance of subsurface features as a reference for other potential clinical applications, such as breast tumor detection, where such deterioration may affect clinical decisions.

Methods S1 3D-mDOI DEMO Instruction

Datasets and codes are available for download at the link below:
https://drive.google.com/drive/folders/1tRjf_DYPRaqLO1mZGGfllIcOqIzZKGPR?usp=share_link

Task 1: Synthetic Phantom Generation
Objective: Generate a synthetic dataset for testing, including the creation of 3D trajectories of simulated photons.
Steps:
1. Run **generate_phantom.m** to produce **_H.mci** and **_T.bin** files for initiating Monte Carlo simulations.
2. Use **batch_process.py** to call **mcxyz.exec** for MAC, generating reflectance data essential for Diffuse Optical Imaging.
3. Convert reflectance data to **.mat** files with **convert_ryx2mat.m** for further testing.

Environment Requirements:
- MATLAB_R2017a for **.m** files execution.
- Python 3.6 environment with dependencies for **batch_process.py**.
- Sufficient memory allocation for **mcxyz.exec** to store 3D photon trajectories.

Task 2: Synthetic Phantom Reconstruction
Objective: Conduct synthetic phantom reconstruction utilizing simulated data to create accurate 3D models for research and analysis.
Steps:
1. Setup Preparation:
    - Ensure that **compute_3dmdoi.py** has access to **dermis_pdf.mat** and **dermis_weight.mat**.
    - If **correction_mua.npz** and **correction_mus.npz** are not available, generate these files with tag “pure” before proceeding with other cases.
2. Simulated Data Utilization:
    - Navigate to the **simulated_data** folder, which includes three cases: pure, shallow, and pigment. Each serves a unique purpose:
        - Pure: For correction generation.
        - Shallow: Simulates pigment penetration up to 1mm.
        - Pigment: Simulates deeper pigment penetration up to 3mm.
3. Environment Configuration:
    - Set up the working environment using the **requirements.txt** file.
    - Adjust compute_3dmdoi.py (line 585) by setting **save_tag** to "pure", "pigment", or "shallow" based on the test case.
4. Execution of codes:
    - Utilize **compute_3dmdoi.py** to perform 3D-mDOI computation, producing mua.npz and mus.npz.
    - Utilize **converter3D.py** to convert mua.npz files into TIFFs for further processing and visualization through Imaris viewer.
    - Perform quantitative analysis using **quantitative_measurement.py**, reproducing measurements as detailed in the project's main draft.

Environment Requirements:
- MATLAB: Essential for handling .mat files.
- Python Environment: Prepare as specified in **requirements.txt** for script execution.

- Simulated Data: Must be organized according to guidelines for **compute_3dmdoi.py**.

This structured approach ensures the synthetic phantom reconstruction is executed efficiently, leveraging simulated data to produce detailed 3D models for further research and analysis.

Task 3: Physical Phantom Reconstruction
Objective: Conduct physical phantom reconstruction by employing detailed imaging and analysis techniques to create accurate 3D models.

Steps:

1. Setup Preparation:
   - Verify the presence of **pdf_map.mat**, **w_map.mat**, and **correction.mat** within the Matlab **CODE** folder for seamless operation.
   - Gather the required raw image data from the **OPTIMAP_data** folder, which includes **pa_imgs.mat**, **pb_imgs.mat**, **pc_imgs.mat**, **pd_imgs.mat**, and **bg_imgs.mat**. These files contain cells representing reemitted diffuse data at varying exposures for comprehensive analysis.
2. Execution of codes:
   - Initiate the **main.m** file to begin the reconstruction process, which processes the 3D-mDOI pipeline. Analyze the distribution of light spots for detailed feature extraction. The computed mua and mus values are stored in **phantom_result.mat** for subsequent evaluation.
   - Post-reconstruction analysis is supported by **analysis.py**, requiring Python 3.7 environment with dependencies.

3. Result Data Utilization:
   - Maintain a structured directory with **phantom_result.mat**, **fem_result.npz**, and **bg_Ref.mat** in the **result_data** folder for analysis. The **ims_file** folder should contain IMS files for 3D-mDOI, Finite Element Method (FEM), and Ground-truth visualization.

Environment Requirements:

- MATLAB: Utilize MATLAB R2017a or a more recent version, adjusting functions as necessary to ensure compatibility.
- Utilize the **Imaris file converter** to transfer output files into the IMS format, and employ the **Imaris viewer** for visual analysis of the 3D models. For detailed guidance, visit the official Imaris learning pages:
  Imaris file converter: https://imaris.oxinst.com/learning/view/article/importing-data-into-imaris
  Imaris viewer: https://imaris.oxinst.com/imaris-viewer

This framework for physical phantom reconstruction emphasizes the importance of precise data collection and sophisticated analysis to replicate the physical characteristics of phantoms in a 3D model, aiding in the advancement of medical imaging research.

Additional Information
For detailed information on the code and environment setup, refer to the specific README files within each task folder. Ensure proper environment setup and file locations as outlined to facilitate the successful execution of each task.

References

1. S. Arridge *et al.*, Approximation errors and model reduction with an application in optical diffusion tomography. *Inverse problems* **22**, 175 (2006).
2. T. J. Farrell, M. S. Patterson, B. Wilson, A diffusion theory model of spatially resolved, steady-state diffuse reflectance for the noninvasive determination of tissue optical properties in vivo. *Medical physics* **19**, 879-888 (1992).
3. S. Feng, F.-A. Zeng, B. Chance, Photon migration in the presence of a single defect: a perturbation analysis. *Applied optics* **34**, 3826-3837 (1995).
4. I. Gkioulekas, A. Levin, T. Zickler, in *European Conference on Computer Vision*. (Springer, 2016), pp. 685-701.
5. V. C. Kavuri, Z.-J. Lin, F. Tian, H. Liu, Sparsity enhanced spatial resolution and depth localization in diffuse optical tomography. *Biomedical Optics Express* **3**, 943-957 (2012).
6. C. K. Hayakawa, J. Spanier, in *Monte Carlo and Quasi-Monte Carlo Methods 2002*. (Springer, 2004), pp. 227-241.
7. A. Garcia-Uribe *et al.*, In-vivo characterization of optical properties of pigmented skin lesions including melanoma using oblique incidence diffuse reflectance spectrometry. *Journal of biomedical optics* **16**, 020501-020501-020503 (2011).
8. E. Alerstam, S. Andersson-Engels, T. Svensson, White Monte Carlo for time-resolved photon migration. *Journal of biomedical optics* **13**, 041304 (2008).
9. L. V. Wang, S. L. Jacques, Source of error in calculation of optical diffuse reflectance from turbid media using diffusion theory. *Computer methods and programs in biomedicine* **61**, 163-170 (2000).
10. L. Wang, S. L. Jacques, Hybrid model of Monte Carlo simulation and diffusion theory for light reflectance by turbid media. *JOSA A* **10**, 1746-1752 (1993).
11. X. Zhong, X. Wen, D. Zhu, Lookup-table-based inverse model for human skin reflectance spectroscopy: two-layered Monte Carlo simulations and experiments. *Optics express* **22**, 1852-1864 (2014).
12. M. Doulgerakis-Kontoudis, A. T. Eggebrecht, S. Wojtkiewicz, J. P. Culver, H. Dehghani, Toward real-time diffuse optical tomography: accelerating light propagation modeling employing parallel computing on GPU and CPU. *Journal of biomedical optics* **22**, 125001 (2017).
13. D. Schadendorf *et al.*, Melanoma. *The Lancet* **392**, 971-984 (2018).
14. F. Ayers, A. Grant, D. Kuo, D. J. Cuccia, A. J. Durkin, in *Design and Performance Validation of Phantoms Used in Conjunction with Optical Measurements of Tissue*. (International Society for Optics and Photonics, 2008), vol. 6870, pp. 687007.
